# Efficacy and safety of ALA/MLA photodynamic therapy for superficial and nodular basal cell carcinoma: a systematic review and meta-analysis

**DOI:** 10.3389/fonc.2026.1802984

**Published:** 2026-04-14

**Authors:** Meiyu Jiang, Deyu Li, Ye Huang, Qiuhua Zhang

**Affiliations:** 1 Department of Dermatology, Yantaishan Hospital, Yantai, Shandong, China; 2 Department of Dermatology, Zhejiang Provincial People's Hospital Bijie Hospital (The First People's Hospital of Bijie), Bijie, Guizhou, China; 3 Department of Dermatology, People’s Hospital of Honghuagang District, Zunyi, Guizhou, China; 4 Department of General Practice, Hunan University of Medicine General Hospital, Huaihua, Hunan, China

**Keywords:** 5-aminolevulinic acid, basal cell carcinoma, meta-analysis, methyl aminolevulinate, nodular basal cell carcinoma, photodynamic therapy, superficial basal cell carcinoma, systematic review

## Abstract

**Background:**

Basal cell carcinoma (BCC) is the most prevalent non-melanoma skin cancer, with surgical excision as the gold standard—though it carries risks of cosmetic scarring and functional impairment. Photodynamic therapy (PDT) using 5-aminolevulinic acid (ALA) or methyl aminolevulinate (MAL) emerges as a non-invasive alternative, yet evidence on its relative efficacy across superficial (sBCC) and nodular (nBCC) subtypes remains inconsistent.

**Methods:**

This systematic review and meta-analysis adhered to PRISMA 2020 guidelines, with a comprehensive search of PubMed, Embase, Cochrane Library, and Web of Science up to December 31, 2025. Eligible studies included randomized controlled trials (RCTs) and single-arm studies evaluating ALA-PDT/MLA-PDT for histologically confirmed sBCC or nBCC. Study quality was assessed via the Methodological Index for Non-Randomized Studies (MINORS) scale, and statistical analyses were performed using Review Manager 5.4.

**Results:**

A comprehensive literature search identified 3832 records, with 55 eligible studies (41 for sBCC, 34 for nBCC) ultimately included, involving 2123 patients and 2995 lesions. For sBCC, the pooled complete response (CR) rate was 0.88 (95% CI: 0.85–0.91, p<0.00001) with high heterogeneity (I²=87%); no significant difference in CR rate was observed between ALA-PDT (0.87) and MAL-PDT (0.90) (p=0.47), and BF-200 ALA-PDT achieved a CR rate of 0.90. The pooled beauty effect rate for sBCC was 0.91 (95% CI: 0.87–0.95, p<0.00001), with a low pooled adverse event incidence of 0.08 (95% CI: 0.03–0.14, p=0.004) and a pooled recurrence rate of 0.13 (95% CI: 0.09–0.18, p<0.00001). For nBCC, the pooled CR rate was 0.75 (95% CI: 0.70–0.80, p<0.00001, I²=92%), with MAL-PDT (0.78) showing a statistically significant higher CR rate than ALA-PDT (0.69) (p=0.04); BF-200 ALA-PDT and AFL-MAL-PDT achieved CR rates of 0.89 and 0.84, respectively. The pooled beauty effect rate for nBCC was 0.90 (95% CI: 0.83–0.96, p<0.00001), with a pooled recurrence rate of 0.15 (95% CI: 0.10–0.20, p<0.00001); adverse event data were insufficient for pooling, with individual studies reporting mild, manageable local reactions. Subgroup analyses revealed that study design, light dose, and number of treatment sessions were the main factors contributing to heterogeneity in key outcomes.

**Conclusions:**

ALA/MAL-PDT is an effective and safe non-invasive therapeutic option for both sBCC and nBCC, with excellent cosmetic outcomes for both subtypes. MAL-PDT exhibits significantly superior efficacy in nBCC compared with ALA-PDT, while the two photosensitizers show comparable therapeutic effects in sBCC. Novel PDT formulations including BF-200 ALA-PDT and AFL-MAL-PDT demonstrate promising CR rates for BCC, providing new treatment alternatives for clinical practice. Standardization of treatment parameters (e.g., light dose, treatment sessions) and differentiation of study design types can effectively reduce heterogeneity in PDT efficacy evaluation, and ALA/MAL-PDT should be prioritized for patients seeking minimally invasive treatment, those with multiple lesions, or those with contraindications to surgical excision.

## Introduction

Basal cell carcinoma (BCC) is the most prevalent non-melanoma skin cancer globally, accounting for approximately 80% of all cutaneous malignancies, with a steadily rising incidence driven by cumulative ultraviolet radiation exposure, aging populations, and immunosuppression (1, 2). While surgical excision remains the gold standard for BCC treatment due to its high cure rate (>95%), it is associated with inherent limitations such as cosmetic scarring, functional impairment in cosmetically sensitive areas, and increased morbidity in patients with multiple lesions or comorbidities that contraindicate surgery (3, 4). Radiotherapy and topical therapies offer alternative options but are limited by long-term side effects (radiation-induced skin changes) or variable response rates, particularly in thicker or aggressive subtypes (5, 6).

Photodynamic therapy (PDT) has emerged as a promising non-invasive treatment for BCC, leveraging the selective accumulation of photosensitizers in neoplastic cells and subsequent activation by visible light to generate reactive oxygen species that induce tumor cell death (7). 5-aminolevulinic acid (ALA) and its methyl ester (methyl aminolevulinate, MAL) are the most widely used topical photosensitizers in BCC treatment, with advantages including minimal damage to surrounding healthy tissue, excellent cosmetic outcomes, and repeatability (8, 9). However, clinical evidence regarding the relative efficacy of ALA-PDT and MAL-PDT across different BCC subtypes (superficial, sBCC; nodular, nBCC) remains inconsistent. Previous studies have reported higher complete response (CR) rates for sBCC compared to nBCC, attributed to the deeper invasive nature of nodular lesions limiting photosensitizer penetration and light distribution (10, 11). Additionally, variations in key treatment parameters such as optical wavelength, dosage, and delivery modality, as well as study design, have hindered a comprehensive understanding of PDT’s true therapeutic potential (12, 13).

Despite several systematic reviews and meta-analyses focusing on PDT for BCC (14–16), most have either limited their scope to a single photosensitizer, excluded specific BCC subtypes, or failed to incorporate recent clinical trials evaluating novel formulations (e.g., BF-200 ALA, AFL-MAL) (17, 18). A robust synthesis of evidence is urgently needed to clarify the efficacy of ALA-PDT versus MAL-PDT in treating sBCC and nBCC, identify the optimal treatment parameters, explore factors influencing treatment response, and assess long-term outcomes and safety profiles. Therefore, this meta-analysis aims to systematically aggregate data from randomized controlled trials (RCTs) and single-arm studies to provide a comprehensive evaluation of ALA/MLA-PDT for sBCC and nBCC, offering evidence-based guidance for clinical practice.

## Materials and methods

### Study registration

This systematic review and meta-analysis adheres strictly to the Preferred Reporting Items for Systematic Reviews and Meta-Analyses (PRISMA) 2020 guidelines to ensure methodological rigor, transparency, and reproducibility of the research process (19), and the study protocol has been prospectively registered in the International Prospective Register of Systematic Reviews (PROSPERO) under the registration number CRD420261300183.

### Eligibility criteria

Eligible studies include randomized controlled trials (RCTs), prospective cohort studies, retrospective cohort studies, or single-arm clinical studies with clear descriptions of the study population, intervention protocols, and outcome measures (conference abstracts and unpublished data are considered if sufficient detail for data extraction and quality assessment is provided); the study population must be adult patients (aged ≥18 years) with histologically confirmed sBCC or nBCC, with no restrictions on gender, ethnicity, geographical region, or disease stage as long as the tumor is classified as either superficial or nodular subtype; the intervention must involve ALA-PDT or MLA-PDT as monotherapy (studies involving combination therapies are excluded unless data for PDT monotherapy can be extracted independently); and outcome measures must include at least one of CR rate, PR rate, Beauty Effect Rate (defined as the proportion of patients with excellent or good cosmetic outcomes evaluated by both clinicians and patients based on the criteria of no obvious scarring, pigmentation abnormality, or skin texture change at the lesion site after treatment, consistent with clinical practice guidelines), 1-year Survival Rate, 3-year Survival Rate, 5-year Survival Rate, Recurrence Rate, or Incidence Rate of Adverse Events; excluded studies are non-human studies, those focusing on other types of skin cancers or non-superficial/non-nodular subtypes of basal cell carcinoma, studies with incomplete or unextractable outcome data, those with significant methodological flaws, duplicate publications or overlapping patient cohorts (only the most comprehensive or latest study is included), and non-English language publications to ensure consistent data interpretation and extraction.

### Information sources and search strategy

A comprehensive literature search will be conducted across four major electronic databases (PubMed, Embase, the Cochrane Library, Web of science) from the inception of each database up to December 31, 2025; a combination of Medical Subject Headings (MeSH) terms, Emtree terms, and free-text keywords will be utilized to maximize search sensitivity and specificity, with core terms including “ALA-PDT”, “MLA-PDT”, “photodynamic therapy”, “basal cell carcinoma”, “superficial basal cell carcinoma”, and “nodular basal cell carcinoma”, combined using Boolean operators “AND” and “OR” to form a logical search string (e.g., (“ALA-PDT” OR “MLA-PDT” OR “photodynamic therapy”) AND (“basal cell carcinoma” OR “superficial basal cell carcinoma” OR “nodular basal cell carcinoma”)), and study design-related terms such as “clinical trial”, “prospective study”, “retrospective study”, and “single-arm study” will be incorporated to refine results; the search will be restricted to English-language publications involving human subjects, and reference lists of all retrieved articles and relevant systematic reviews will be manually screened to identify potentially eligible studies missed in the initial database search.

### Study selection

The study selection process will be performed independently by two reviewers using a standardized screening tool: all retrieved citations will first be imported into EndNote X9 software to remove duplicates, followed by title and abstract screening by the two reviewers to exclude studies that clearly do not meet inclusion criteria, full-text articles of potentially eligible studies will be retrieved for detailed assessment against predefined eligibility criteria, any discrepancies between reviewers during screening will be resolved through face-to-face discussion and consensus (with consultation of a third independent reviewer if consensus cannot be reached), and the entire screening process will be documented using a PRISMA flow diagram, including the number of studies identified, excluded, and ultimately included, along with reasons for exclusion.

### Data collection and data items

A pre-designed, standardized data extraction form will be used to collect relevant information from each included study, with data extraction performed independently by two reviewers and cross-checked for accuracy; extracted data include study characteristics (first author, publication year, country/region of study, study design), patient demographics (number of lesions), intervention details (type of PDT, light source parameters, and number of treatment sessions), and outcome data (for each predefined outcome indicator, the number of events and total number of evaluable patients, with specific definitions for each outcome); if required data are missing or unclear, corresponding authors of original studies will be contacted via email to request additional information, and if no response is received within 4 weeks, the study will be included only if available data are sufficient for analysis, otherwise excluded.

### Risk-of-bias assessment

The quality of included studies will be assessed independently by two reviewers using the Methodological Index for MINORS scale: for non-comparative studies (the majority expected for single-arm rate meta-analysis), the 8-item MINORS scale will be used (each item scored 0-2, maximum total score 16), with studies scoring ≥12 classified as high quality, 9–11 as moderate quality, and <8 as low quality; any discrepancies in quality assessment will be resolved through discussion between the two reviewers or consultation of a third reviewer, and results of quality assessment will be presented in a tabular format.

### Statistical analysis

Statistical analyses will be performed using Review Manager 5.4 software; given the dichotomous nature of outcome indicators (expressed as rates), a single-arm rate meta-analysis method will be employed to pool effect sizes, with pooled rates and their 95% confidence intervals (CIs) calculated using the generic inverse variance method, and weights assigned based on study sample size (inverse variance weighting) to account for differences in study precision; heterogeneity among studies will be evaluated using the Cochran Q test (p<0.05 indicating significant heterogeneity) and I² statistic (I² <25% for low heterogeneity, 25%-50% for moderate heterogeneity, >50% for high heterogeneity), with a fixed-effects model used for low to moderate heterogeneity (I² ≤50%) and a random-effects model for high heterogeneity (I² >50%) to account for between-study variability; subgroup analyses will be conducted based on factors such as the type of photodynamic therapy (ALA-PDT and MLA-PDT), tumor subtype (superficial type and nodular type), study design (real-world studies and randomized controlled trials), as well as the number of treatments, light dose, and follow-up duration; a p-value <0.05 will be considered statistically significant for all analyses except heterogeneity tests.

## Results

### Search and screening results

A comprehensive literature search was conducted across four major electronic databases from their inception up to December 31, 2025, yielding a total of 3832 initial records (845 from PubMed, 1869 from Embase, 46 from Cochrane Library, and 1072 from Web of Science).

After importing all retrieved citations into EndNote X9 software for duplicate removal, 1726 duplicate records were excluded, leaving 2106 records for initial title and abstract screening. During this screening phase, 1912 records were excluded for failing to meet the preliminary eligibility criteria, resulting in 194 reports selected for full-text retrieval; however, 57 of these reports could not be retrieved, leaving 137 reports for detailed eligibility assessment.

**Figure 1:**
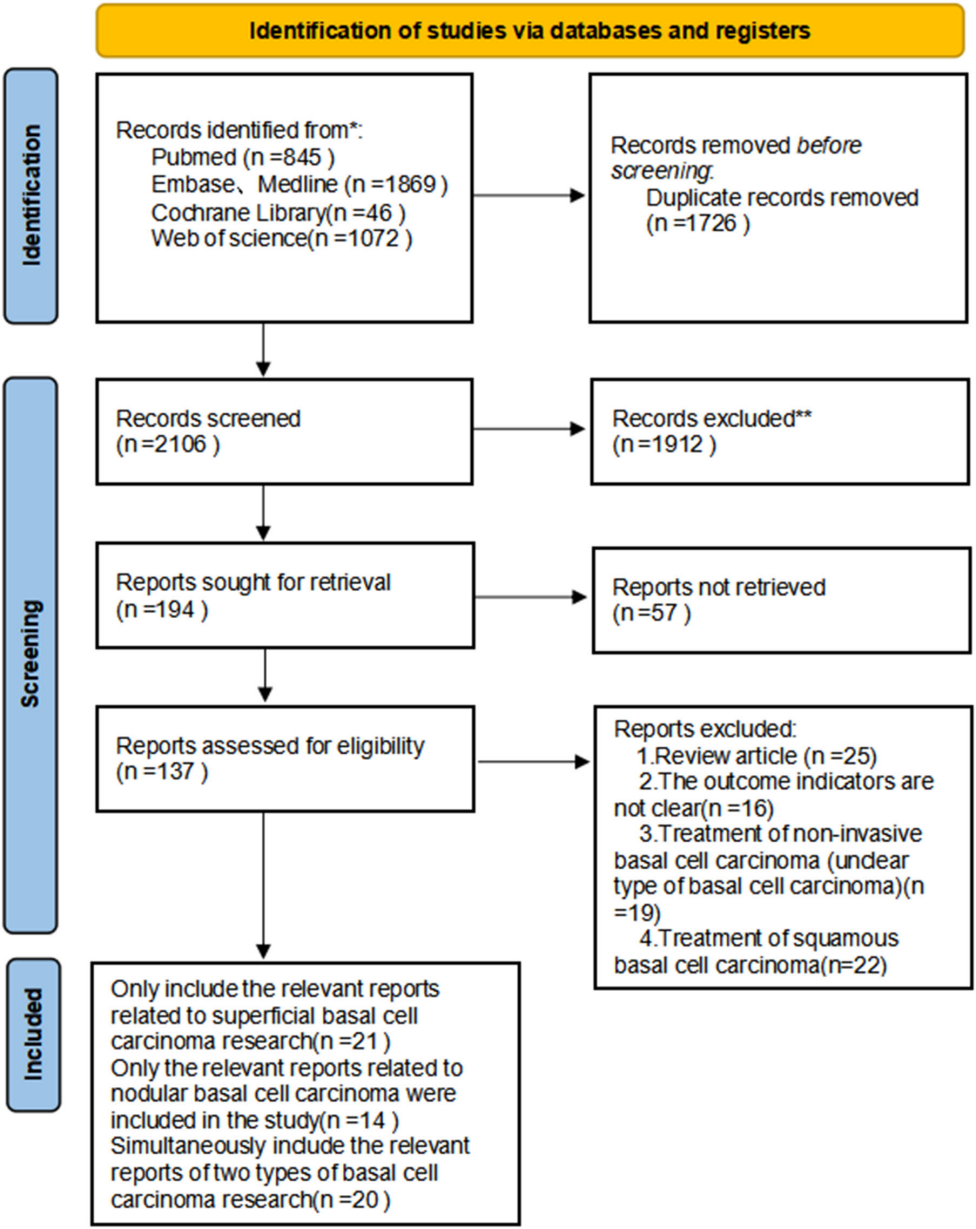
Preferred Reporting Items for Systematic Reviews and Meta-Analyses (PRISMA) diagram of the study selection process for ALA/MLA photodynamic therapy in the treatment of superficial and nodular basal cell carcinoma.

The full-text assessment led to the exclusion of 123 reports based on predefined criteria: 25 were review articles, 22 focused on basal cell carcinoma with unclear subtypes, 16 lacked relevant outcome indicators, 21 addressed the treatment of non-invasive or squamous cell carcinoma, and 20 were studies simultaneously involving both superficial and nodular basal cell carcinoma without extractable separate data for each subtype (**Figure 1**).

Ultimately, 41 studies focusing on sBCC were included in the meta-analysis, with these studies originating from multiple countries including Canada, Austria, the UK, Sweden, Italy, the Netherlands, Poland, China, Spain, Russia, and the United States. The included sBCC studies consisted of 27 single-arm studies (7, 8, 20–44) and 14 RCTs (10, 12, 45–56), involving a total of 1453 lesions and 987 patients, with treatment interventions including 20% ALA-PDT, 10% ALA-PDT, BF-200 ALA-PDT, and MAL-PDT (methyl aminolaevulinate PDT), treatment cycles ranging from 1 to 12, and light parameters varying in wavelength (515–720 nm), intensity (20–350 mW/cm²), and dose (20–350 J/cm²), with evaluation times spanning 1 month to 5 years.

Additionally, 34 studies focusing on nBCC were included, originating from countries such as Austria, Sweden, Italy, Norway, the Netherlands, the UK, Poland, China, South Korea, Brazil, and the United States. These nBCC studies comprised 22 single-arm studies (8, 21–23, 25, 29, 31–33, 35–39, 44, 57–61) and 12 RCTs (11, 13, 45, 46, 48, 51, 53, 62–66), encompassing 1542 lesions and 1136 patients, with similar intervention types to sBCC studies (20% ALA-PDT, MAL-PDT, and AFL-MAL-PDT), treatment cycles of 1 to 4, light parameters ranging in wavelength (417–980 nm), intensity (10–200 mW/cm²), and dose (37–150 J/cm²), and evaluation times from 1 month to 5 years. Key characteristics of the included studies, such as author, publication year, country, study design, treatment details, and outcome data, are summarized in **Table 1** (for sBCC) and **Table 2** (for nBCC).

**Table 1:** The main characteristics included in the study on treating superficial basal cell carcinoma.

Author year	Country	Study design	Number of cycles	Light intensity, and dose	Evaluation time	Intervention	Number of lesions	No. of patient	Age	Gender (Male: Female)	CR	PR	Beauty effect rate	One-year survival rate	Three-years survival rate	Five-years survival rate	Recurrent rate	Incidence rate of adverse events
J.c.KENNEDY 1990 (7)	Canada	single-arm	1	150 - 300 mW/cm^2^	2-3 months	20% ALA-PDT	80	N	N	N	72/80	6/80	N	N	N	N	N	N
Peter Wolf 1992 (8)	Austria	single-arm	1	100 mW/cm^2^	1-2 months	20% ALA-PDT	37	13	N	10/3	36/37	1/37	N	N	N	N	N	N
F. Cairnduffl 1994 (20)	UK	single-arm	1	630 nm,150 mW/cm^2^	2 months	20% ALA-PDT	16	14	N	N	14/16	N	N	N	N	N	N	N
K. SVANBERG 1994 (21)	Sweden	single-arm	1	110 mW/cm^2^	The 1st, 3rd and 12th weeks	20% ALA-PDT	55	21	71(35-90)	14/7	55/55	N	50/55	N	N	N	N	N
Pier G. Calzavara-Pinton 1995 (22)	Italy	single-arm	1-2	630 nm,100 mW/cm^2^	1 months	20% ALA-PDT	23	N	65(24-87)	N	20/23	N	N	N	N	N	2/12	N
S. FIJAN. H. HONIGSMANN 1995 (23)	Austria	single-arm	1	50 - 300 mW/cm^2^	3-20 months	20% ALA-PDT	34	32	N	N	30/34	N	N	N	N	N	N	N
P. J. N. MEIJNDERS 1996 (24)	The Netherlands	single-arm	1	620-650 nm ,50-100 J /cm^2^	1-39 months	20% ALA-PDT	42	16	65(35-89)	N	33/42	8/42	N	N	N	N	1/42	N
A. M. Wennberg 1996 (25)	Sweden	single-arm	1	620-670 nm,166 mW/cm^2^,75/100 J/cm^2^	3 months	20% ALA-PDT	190	37	65( 28-83)	N	144/157	N	144/157	N	N	N	N	N
Regina Fink-Puches 1998 (26)	Austria	single-arm	1	515/570/610 nm	3-60 months	20% ALA-PDT	95	47	68(47-90)	18/29	82/95	9/95	N	N	N	N	36/81	N
YORAM HARTH 1998 (27)	Israel	single-arm	1-3	585-720 nm, 150 mW/cm^2^	6-15 months	20% ALA-PDT	34	22	N	N	26/34	N	N	N	N	N	N	N
A. F. Hürlimann 1998 (28)	Switzerland	single-arm	1	200 mW/cm^2^, 240 J/cm^2^	N	10% ALA-PDT	55	19	65(32-93)	14/5	47/55	8/55	N	N	N	N	N	N
A. M. SOLER 2001 (29)	Norway	single-arm	1-4	100 - 180 mW/cm^2^	3-6 months	20% ALA-PDT	131	59	N	N	119/131	N	N	N	N	N	12/131	N
I. WANG 2001 (45)	Sweden	RCT	1	635 nm,80 ±20 mW/cm^2^	4th, 8th week and 3-month follow-up	20% ALA-PDT	22	88	N	44/44	N	N	N	N	N	N	8/21	N
C. Clark 2003 (30)	UK	single-arm	1	580- 720 nm,20-25 mW/cm^2^	Every 3 months	ALA-PDT	87	81	N	N	66/87	N N N	N N N	N N N	N N N	N N N	4/84	N N N
			2								78/87							
			4								84/87							
M. HORN 2003 (31)	Austria	single-arm	1-2	570- 670 nm,50-200 mW /cm^2^,75 J/cm^2^	3 months	MAL-PDT	49	94	68(32-93)	57/37	45/49	N	N	N	N	N	8/36	N
Aleksander Sieron 2004 (32)	Poland	single-arm	2-12	635 nm, 100-125J/cm^2^	N	20% ALA-PDT	41	36	N	N	39/41	1/41	N	N	N	N	1/41	N
C. Vinciullo 2005 (33)	Austria	single-arm	1-2	570-670 nm,75 J/cm^2^	3,12,24 months	MAL-PDT	80	92	N	N	74/80	N	N	N	N	N	N	N
Willem M. STAR 2006 (34)	Netherlands	single-arm	1	45 J/cm^2^	1、3、6、9、12、18、24、36、48、60 months	20% ALA-PDT	86	15	61	8/7	56/67	N	67/76	N	N	N	11/67	N
Peter Schleier PdD 2007 (46)	Germany	RCT	1	120 J/cm^2^	6 months	10% ALA-PDT	72	24	74(42-96)	13/11	44/72	25/72	N	N	N	N	8/72	8/72
ERM de Haas 2008 (35)	The Netherlands	single-arm	1	630 nm, 80 J/cm^2^	12 months	20% ALA-PDT	430	90	58(33-81)	53/37	419/430	N	412/430	N	N	N	N	N
RM Szeimies 2008 (10)	Germany	RCT	2/4	37 J/cm^2^	12 months	MAL-PDT	128	100	64.5±12.7	64/36	107/118	N	77/83	N	N	N	11/118	37/100
F. Fantini 2011 (36)	Italy	single-arm	1-3	630 nm,37 J/cm^2^	6 months	MAL-PDT	116	116	N	N	95/116	N	N	N	N	N	N	N
Qiang Li 2011 (37)	China	single-arm	3	633 nm,113 J/cm^2^	6 months	MAL-PDT	39	N	N	N	38/39	N	N	N	N	N	N	N
Hatinah C. DE VIJLDER 2012 (47)	The Netherlands	RCT	1	630 nm, 80 J/cm^2^	60 months	20% ALA-PDT	573	195	N	N	469/573	N	N	N	N	N	83/573	N
R. Cosgarea 2012 (48)	Romania	RCT	2	37 J/cm^2^	3 months	20% ALA-PDT	31	32	N	N	31/31	N	N	N	N	N	N	N
Aimée H M M Arits 2013 (49)	Netherlands	RCT	2	630 nm,37 J/cm^2^	3、12 months	16% MAL-PDT	N	202	63 (26-87)	N	N	N	116/186	N	N	N	52/196	N
Dora P Ramirez 2014 (38)	Brazil	single-arm	2	40±8 mW/cm^2^	N	MAL-PDT	199	N	N	N	160/199	29/199	N	N	N	N	N	N
Marieke H. Roozeboom 2016 (50)	The Netherlands	RCT	N	N	3 years	MAL-PDT	202	202	N	96/106	N	N	N	N	118/202	N	N	N
M. Tarstedt 2016 (51)	Sweden	RCT	2	37 J/cm^2^	6 months	20% ALA-PDT	25	N	N	N	17/19	N	N	N	N	N	N	N
						MAL-PDT	39	N			24/28	N	N	N	N	N	N	N
Maud H E Jansen 2018 (6)	The Netherlands	RCT	1-2	N	5 years	MAL-PDT	153	153	62 (26-86)	74/79	N	N	137/153	N	N	105/109	N	N
C.A. Morton 2018 (53)	UK	RCT	1-2	635 nm,37 J/cm^2^	5 years	BF-200 ALA-PDT	119	95	N	N	114/119	N	N	N	N	N	N	N
						MAL-PDT	98	83	N	N	95/98	N	N	N	N	N	N	N
Adel Olasz 2018 (39)	Norway	single-arm	3	37 J/cm^2^	6 months	MAL-PDT	70	N	N	N	67/70	N	N	N	N	N	N	N
Sergio Alique-García 2019 (40)	Spain	single-arm	1-2	630 nm,37 J/cm^2^	12 months	BF-200 ALA-PDT	16	22	N	N	15/16	N	N	N	N	N	1/16	N
						MAL-PDT	14	N	N	N	7/14	N	N	N	N	N	2/14	N
K. P. Nguyen 2019 (54)	The Netherlands	RCT	2	20 J/cm^2^,55 J/cm^2^	3 months	MAL-PDT	21	21	N	N	14/21	6/21	N	N	N	N	N	N
E. Filonenko 2020 (41)	Russia	single-arm	3	630 nm,350 J/cm^2^	3 months	12% ALA-PDT	124	82	N	15/67	119/124	N	N	110/119	105/119	N	5/124	N
Ian T. Logan 2020 (42)	United Kingdom	single-arm	2	630 nm,80 mW/cm^2^, 37 J/cm^2^	3 months	MAL-PDT	21	17	N	N	20/21	1/21	20/21	N	N	N	2/21	N
Francisco José Navarro-Triviño 2020 (43)	Spain	single-arm	2	635 nm,37 J/cm^2^	3、6、12 months	BF-200 ALA-PDT	31	31	63.74	12/19	23/31	N	N	N	N	N	N	N
Clara Gomez 2021 (44)	Spain	single-arm	2-3	630 nm,90 J/cm^2^	3 years	MAL-PDT	76	56	N	28/28	73/76	N	75/76	74/76	73/76	N	N	N
Lieke C.J. van Delft 2022 (12)	The Netherlands	RCT	2	630 nm ± 5 nm, 20 J/cm^2^, 50 mW/cm^2^	5 years	20% ALA-PDT	82	82	65.9 (38-85)	40/42	N	N	61/63	75/82	56/82	46/82	N	N
						MAL-PDT	80	80	63.6 (28-83)	35/45	N	N	56/59	69/79	46/79	45/79	N	N
Paulina Szczepanik-Kułak 2024 (55)	Poland	RCT	2	630±5 nm	3 months	ALA-PDT	11	11	N	N	9/11	N	N	N	N	N	N	N
						MAL-PDT	10	10			8/10	N	N	N	N	N	N	N
Todd Schlesinger 2025 (56)	South Carolina	RCT	2/4	635 nm,37 J/cm^2^	5 years	10% ALA-PDT	145	145	62.4±10.8	67/78	120/145	N	129/145	N	N	N	N	N

ALA, Aminolevulinic acid; MAL, Methyl aminolaevulinate; PDT, Photodynamic therapy; N, Not mentioned.

**Table 2:** The main characteristics included in the study on treating nodular basal cell carcinoma.

Author year	Country	Study design	Number of cycles	Light intensity, and dose	Evaluation time	Intervention	Number of lesions	No. of patient	Age	Gender (Male: Female)	CR	PR	Beauty effect rate	One-year survival rate	Three-years survival rate	Five-years survival rate	Recurrent rate	Incidence rate of adverse events
Peter Wolf 1992 (8)	Austria	single-arm	1	100 mW/cm^2^	1-2 months	20% ALA-PDT	10	13	N	10/3	1/10	N	N	N	N	N	N	N
K.SVANBERG1994 (21)	Sweden	single-arm	1	110 mW/cm^2^	The 1st, 3rd and 12th weeks	20% ALA-PDT	25	21	71(35-90)	14/7	16/25	N	15/25	N	N	N	N	N
Pier G. Calzavara-Pinton1995 (22)	Italy	single-arm	1-2	630 nm,100 mW/cm^2^	1 months	20% ALA-PDT	30	N	65(24-87)	N	15/30	N	N	N	N	N	N	N
S.FIJAN. H.HONIGSMANN1995 (23)	Austria	single-arm	1	50 - 300 mW/cm^2^	3-20 months	20% ALA-PDT	22	32	N	N	7/22	N	N	N	N	N	N	N
A. M. Wennberg1996 (25)	Sweden	single-arm	1	620-670 nm,150-200 mW/cm^2^,100 J/cm^2^	3 months	20% ALA-PDT	10	6	74.5(62–75 )	N	2/10	N	N	N	N	N	N	N
ANA MARIA SOLER1999 (57)	Norway	single-arm	1	550-700 nm,166 mW/cm^2^,75/100 J/cm^2^	3-6 months	20% ALA-PDT	119	58	36 ~ 86	N	113/119	N	103/119	N	N	N	6/119	N
M.R.T.M.THISSEN2000 (58)	The Netherlands	single-arm	1	630-635 nm,120 J/cm^2^	3 months	20% ALA-PDT	24	23	N	N	22/24	N	21/22	N	N	N	N	N
A.M.SOLER2001 (29)	Norway	single-arm	1-4	100-180 mW/cm^2^	3-6 months	ALA-PDT	168	59	N	N	150/168	N	N	N	N	N	18/168	N
I.WANG2001 (45)	Sweden	RCT	1	635 nm,80 ±20 mW/cm^2^	4th, 8th week and 3-month follow-up	20% ALA-PDT	25	88	N	44/44	N	N	N	N	N	N	3/23	N
M.HORN2003 (31)	Austria	single-arm	1-2	570- 670 nm,50-200 mW /cm^2^,75 J/cm^2^	3 months	MAL-PDT	45	94	68(32-93)	57/37	45/52	N	N	N	N	N	4/28	N
ALEKSANDR ITKIN2004 (59)	Massachusetts	single-arm	1	417 nm,10mW /cm^2^	8 months	20% ALA-PDT	16	2	N	N	5/16	N	N	N	N	N	N	N
Lesley E. Rhodes2004 (4)	England	single-arm	1-2	570- 670 nm,50-200 mW /cm^2^,75 J/cm^2^	3 months	MAL-PDT	53	52	69 (40-95)	32/20	48/53	N	28/29	N	N	N	10/53	27/52
Aleksander Sieron2004 (32)	Poland	single-arm	2-12	635 nm, 100-125J/cm^2^	N	20% ALA-PDT	35	35	N	N	21/35	10/35	N	N	N	N	7/35	N
C. Vinciullo2005 (33)	Austria	single-arm	1-2	570-670 nm,75 J/cm^2^	3,12,24 months	MAL-PDT	33	36	N	N	27/33	N	N	N	N	N	N	N
Lesley E. Rhodes2007 (4)	England	single-arm	2/4	570-670 nm,75 J/cm^2^	5 years	MAL-PDT	53	50	69(40-95)	N	40/53	N	36/44	N	N	N	7/53	N
Peter Schleier PdD2007 (46)	Germany	RCT	1	120 J/cm^2^	6 months	MAL-PDT	40	24	74(42-96)	13/11	23/40	16/40	N	N	N	N	5/40	5/40
ERM de Haas2008 (35)	The Netherlands	single-arm	1	630 nm, 80 J/cm^2^	12 months	20% ALA-PDT	20	16	59(45-75)	12/4	16/20	N	18/20	N	N	N	N	N
K. Mosterd 2008 (11)	The Netherlands	RCT	1	580-720 nm,100 mW/cm^2^,150 J/cm^2^	3 months	20% ALA-PDT	83	83	N	N	78/83	N	N	N	N	N	N	N
Peter Foley 2009(62)	Australia	RCT	2	570-670 nm,50-200mW/cm^2^,75 J/cm^2^	3/6 months	MAL-PDT	75	66	N	47 / 19	55/75	N	42/43	N	N	N	N	60/66
J. Lippert2010 (60)	Kralovs	single-arm	2	980 nm	6/12 months	MAL-PDT	84	84	N	N	82/84	N	N	N	N	N	N	N
F. Fantini2011 (36)	Italy	single-arm	1-3	630 nm,37 J/cm^2^	6 months	MAL-PDT	78	78	N	N	26/78	N	N	N	N	N	N	N
Qiang Li2011 (37)	China	single-arm	3	633 nm,113 J/cm^2^	6 months	MAL-PDT	28	N	N	N	23/28	N	N	N	N	N	N	N
R. Cosgarea2012 (48)	Romania	RCT	2	37 J/cm^2^	3 months	20% ALA-PDT	17	32	N	N	15/17	N	N	N	N	N	2/17	N
Marieke H. Roozeboom2013 (63)	The Netherlands	RCT	1	150 J/cm^2^	5 years	20% ALA-PDT	83	83	64.0 (24-83)	43/40	N	N	N	N	N	N	23/83	N
Dora P Ramirez2014 (38)	Brazil	single-arm	2	40±8 mW/cm^2^	N	MAL-PDT	151	N	N	N	112/151	37/151	N	N	N	N	N	N
C.S. Haak2014(64)	Denmark	RCT	2	N	12 months	MAL-PDT	16	16	N	N	10/16	N	N	N	N	N	3/16	N
S.H. Choi2016 (65)	South Korea	RCT	1	632 nm,37 J/cm^2^	3 months	AFL-MAL-PDT	19	18	N N	N N	16/19	N	N	N	N	N	1/19	N
			2			MAL-PDT	18	16			9/18	N	N	N	N	N	10/18	N
M. Tarstedt 2016 (51)	Sweden	RCT	2	37 J/cm^2^	6 months	20% ALA-PDT	19	N	N	N	15/18	N	N	N	N	N	N	N
C.A. Morton2018 (53)	UK	RCT	1-2	635 nm,37 J/cm^2^	5 years	BF-200 ALA-PDT	28	21	N	N	25/28	N	N	N	N	N	N	N
						MAL-PDT	28	21	N	N	21/28	N	N	N	N	N	N	N
Adel Olasz 2018 (39)	Norway	single-arm	3	37 J/cm^2^	6 months	MAL-PDT	190	N	N	N	172/190	N	N	N	N	N	N	N
Clara Gomez 2021 (44)	Spain	single-arm	2-3	630 nm,90 J/cm^2^	3 years	MAL-PDT	144	118	N	61/57	137/144	N	144/144	140/144	137/144	N	N	N
Ana Gabriela Salvio2024 (61)	Brazil	single-arm	1	150 J/cm^2^	1 months	MAL-PDT	96	52	N	N	86/96	N	N	N	N	N	N	N
Ana Gabriela Salvio2025 (13)	Brazil	RCT	1	630 nm,125 mW/cm^2^,150 J/cm^2^	1 months	20% ALA-PDT	189	189	64.2	N	171/189	N	N	N	N	149/189	N	N
						MAL-PDT	189	189	65	N	161/187	N	N	N	N	138/189	N	N
Leore Lavin2025 (66)	UK	RCT	2	633 nm,37 J/cm^2^	1-3 months	20% ALA-PDT	13	11	N	N	10/13	N	N	N	N	N	N	N

ALA, Aminolevulinic acid; MAL, Methyl aminolaevulinate; PDT, Photodynamic therapy; N, Not mentioned.

### Quality assessment

The quality of all 55 included studies was independently assessed by two reviewers using the 8-item MINORS scale (scored 0–2 per item, maximum 16 points), with discrepancies resolved via discussion or third reviewer consultation. Overall, 38 studies (82.6%) were classified as high-quality, and 8 as moderate-quality, with no low-quality studies identified; nearly all studies scored full points for clear aims, consecutive patient inclusion, and prospective data collection, while minor deficiencies were noted in follow-up attrition reporting and pre-specified sample size calculation in a small subset of studies. Detailed scores are summarized in **Supplementary Table 1**.

### Superficial basal cell carcinoma

A total of 41 studies focusing on sBCC were included, involving 987 patients and 1453 lesions, with interventions including ALA-PDT (20%, 10%, BF-200), MAL-PDT, and varying light parameters and treatment cycles. The meta-analysis results showed significant therapeutic effects of PDT for sBCC across all predefined outcome indicators, with some heterogeneity observed among studies.

**Figure 2:**
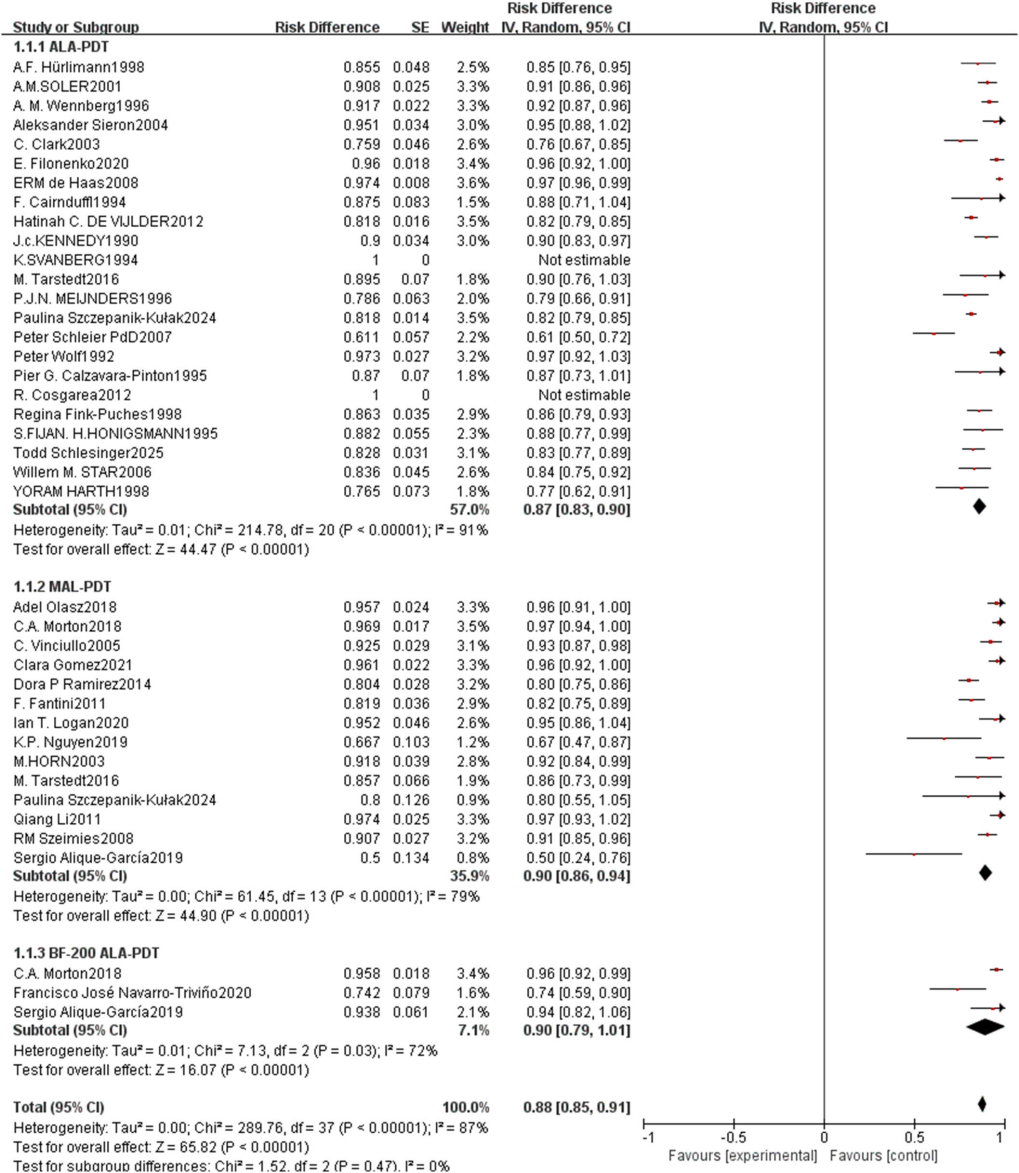
Forest plot of pooled CR rate of ALA/MLA photodynamic therapy for sBCC.

**Figure 3:**
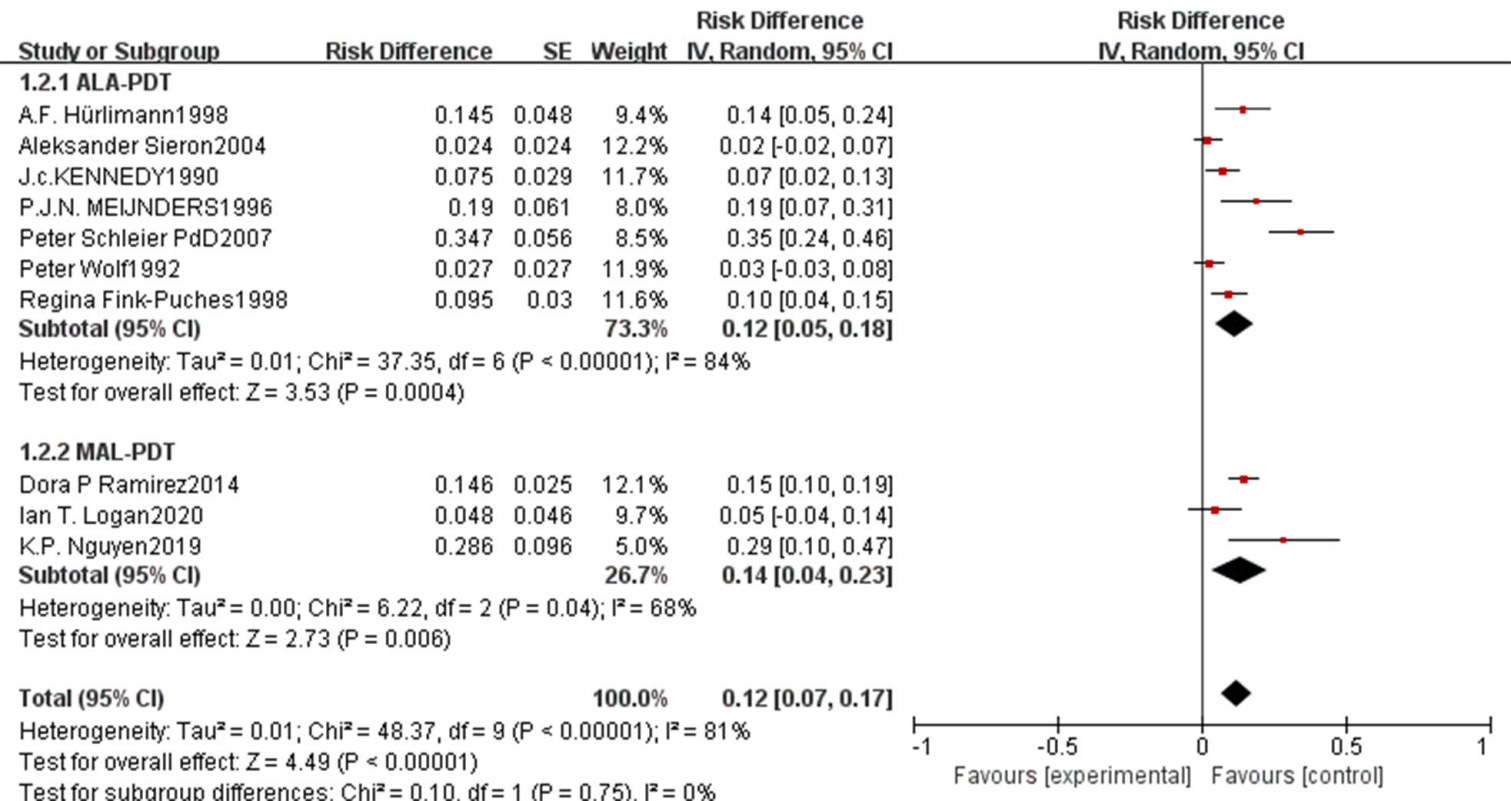
Forest plot of pooled PR rate of ALA/MLA photodynamic therapy for sBCC.

For CR rate, the pooled rate across all included studies was 0.88 (95% CI: 0.85–0.91, p<0.00001, **Figure 2**) with high heterogeneity (I²=87%). Subgroup analysis by PDT type revealed that ALA-PDT achieved a pooled CR rate of 0.87 (95% CI: 0.83–0.90), MAL-PDT of 0.90 (95% CI: 0.86–0.94), and BF-200 ALA-PDT of 0.90 (95% CI: 0.79–1.01), with no statistically significant difference between subgroups (p=0.47, I²=0%). The PR rate had a pooled value of 0.12 (95% CI: 0.07–0.17, p<0.00001, **Figure 3**) and high heterogeneity (I²=81%), with ALA-PDT subgroup at 0.12 (95% CI: 0.05–0.18) and MAL-PDT subgroup at 0.14 (95% CI: 0.04–0.23), and no significant subgroup difference (p=0.50, I²=0%).

**Figure 4:**
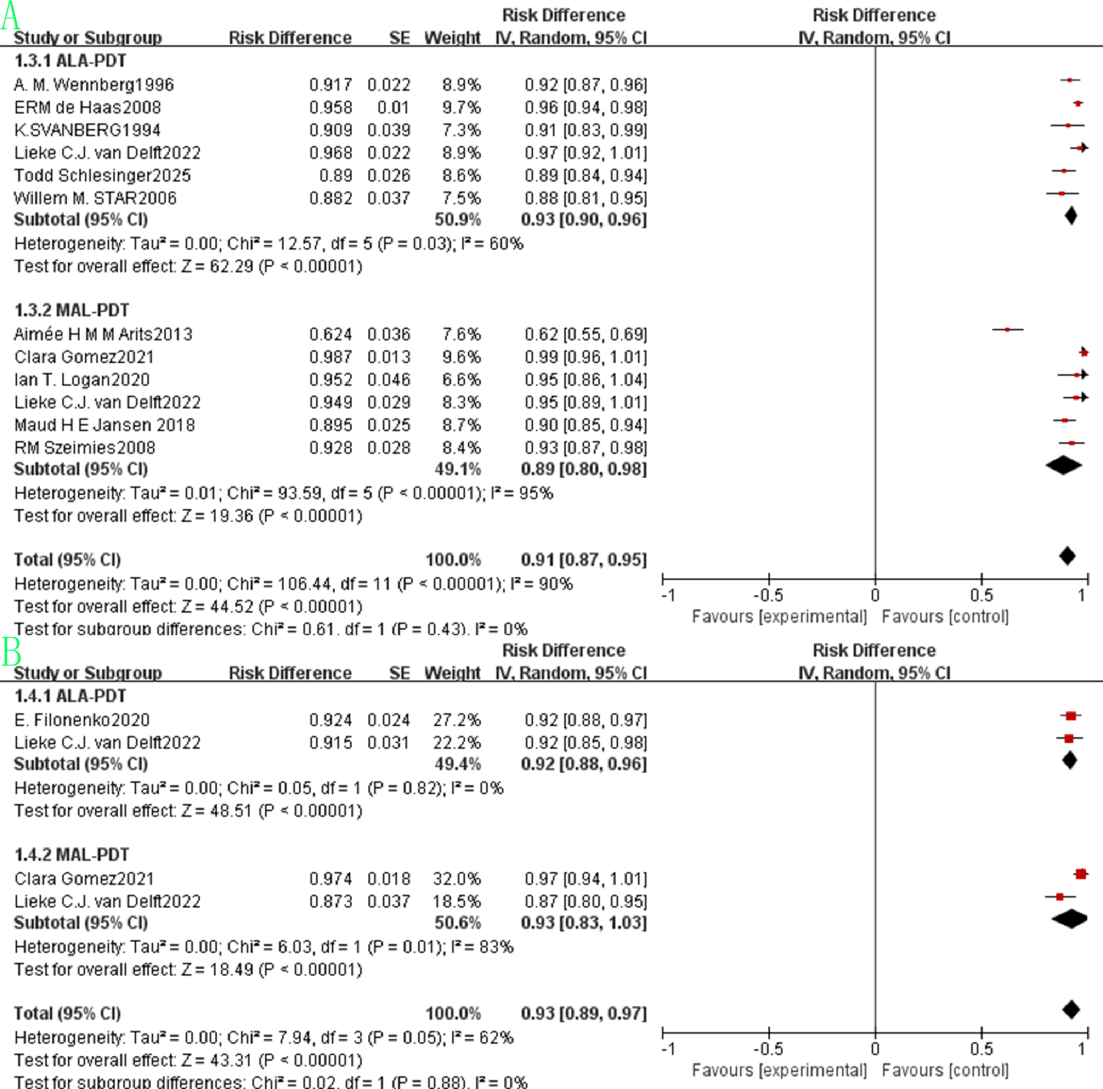
Forest plot of pooled beauty effect rate **(A)** and 1-year survival rate **(B)** of ALA/MLA photodynamic therapy for sBCC.

**Figure 5:**
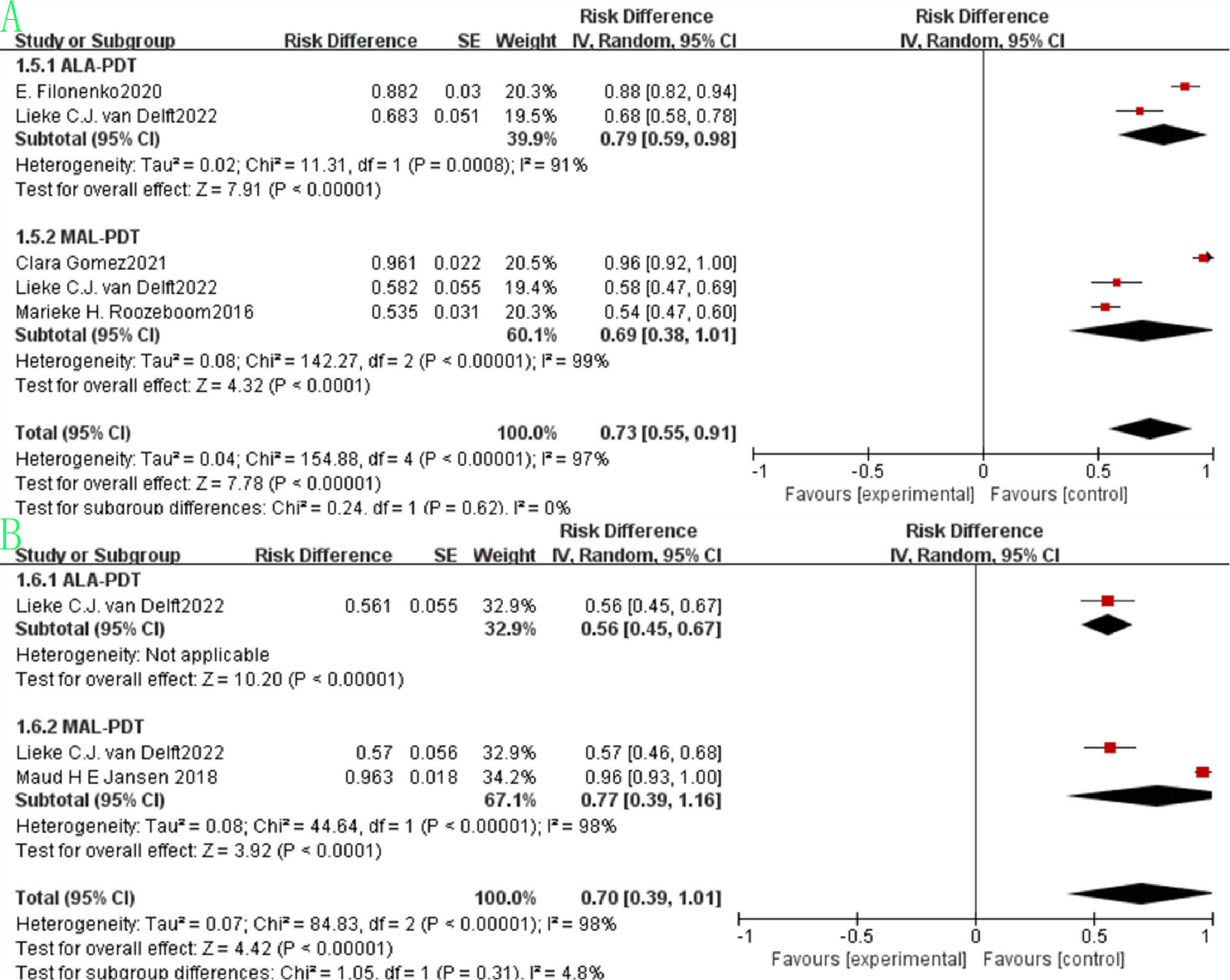
Forest plot of pooled 3-year survival rate **(A)** and 5-year survival rate **(B)** of ALA/MLA photodynamic therapy for sBCC.

The pooled beauty effect rate was 0.91 (95% CI: 0.87–0.95, p<0.00001, **Figure 4A**) with substantial heterogeneity (I²=90%). ALA-PDT showed a slightly higher pooled rate of 0.93 (95% CI: 0.90–0.96) compared to MAL-PDT’s 0.89 (95% CI: 0.80–0.98), but the difference between subgroups was not statistically significant (p=0.43, I²=0%). Regarding survival outcomes, the pooled 1-year survival rate was 0.93 (95% CI: 0.89–0.97, p<0.00001, **Figure 4B**) with moderate heterogeneity (I²=62%), where ALA-PDT and MAL-PDT subgroups had pooled rates of 0.92 (95% CI: 0.88–0.96) and 0.93 (95% CI: 0.83–1.03), respectively (p=0.88, I²=0%). The pooled 3-year survival rate was 0.73 (95% CI: 0.55–0.91, p<0.00001, **Figure 5A**) with extremely high heterogeneity (I²=97%), with ALA-PDT at 0.68 (95% CI: 0.58–0.78) and MAL-PDT at 0.69 (95% CI: 0.38–1.01) (p=0.62, I²=0%). The pooled 5-year survival rate was 0.70 (95% CI: 0.39–1.01, p<0.00001, **Figure 5B**) with very high heterogeneity (I²=98%), where ALA-PDT subgroup was 0.56 (95% CI: 0.45–0.67) and MAL-PDT subgroup was 0.77 (95% CI: 0.39–1.16) (p=0.31, I²=44%).

**Figure 6:**
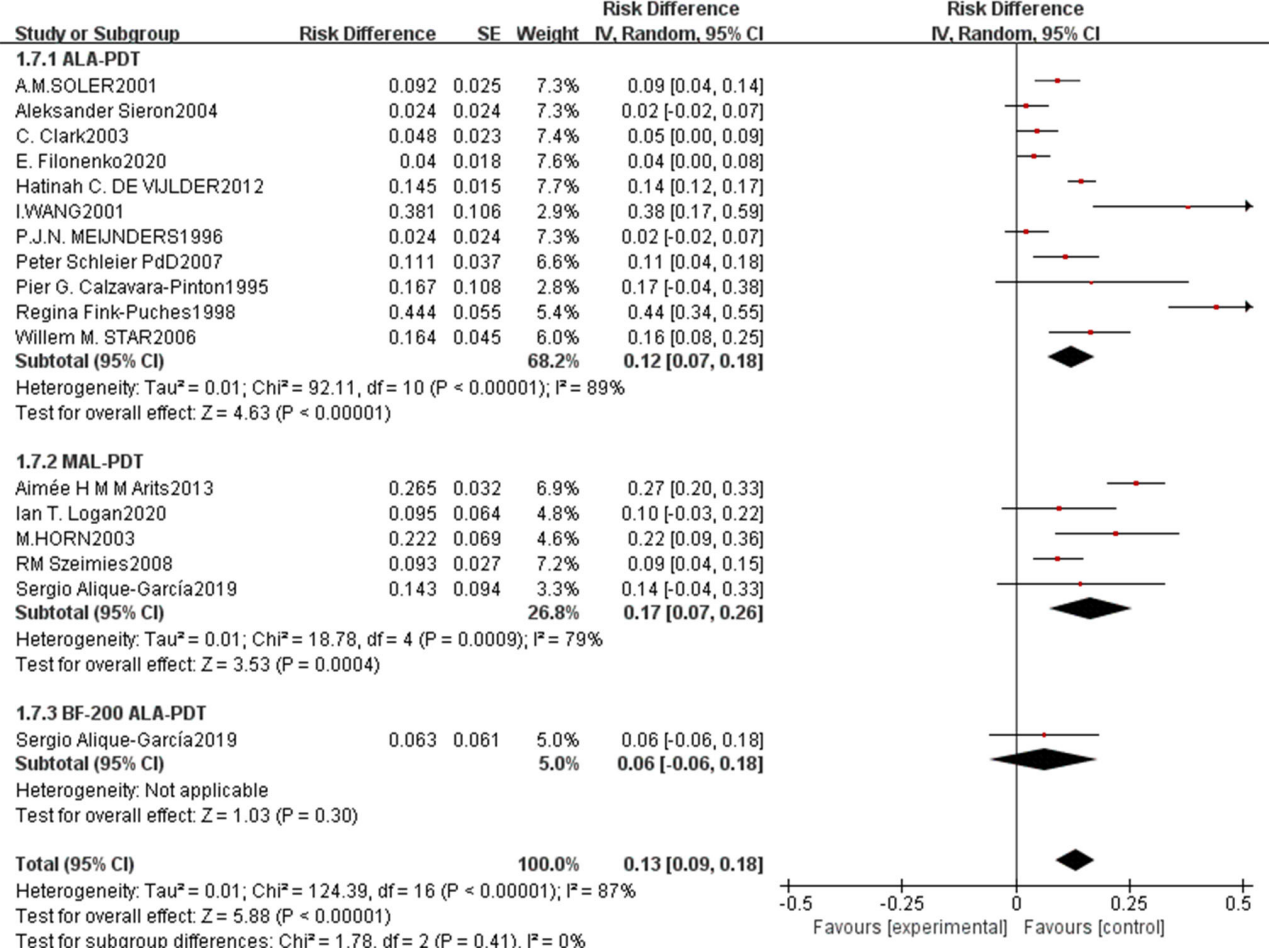
Forest plot of pooled recurrence rate of ALA/MLA photodynamic therapy for sBCC.

**Figure 7:**
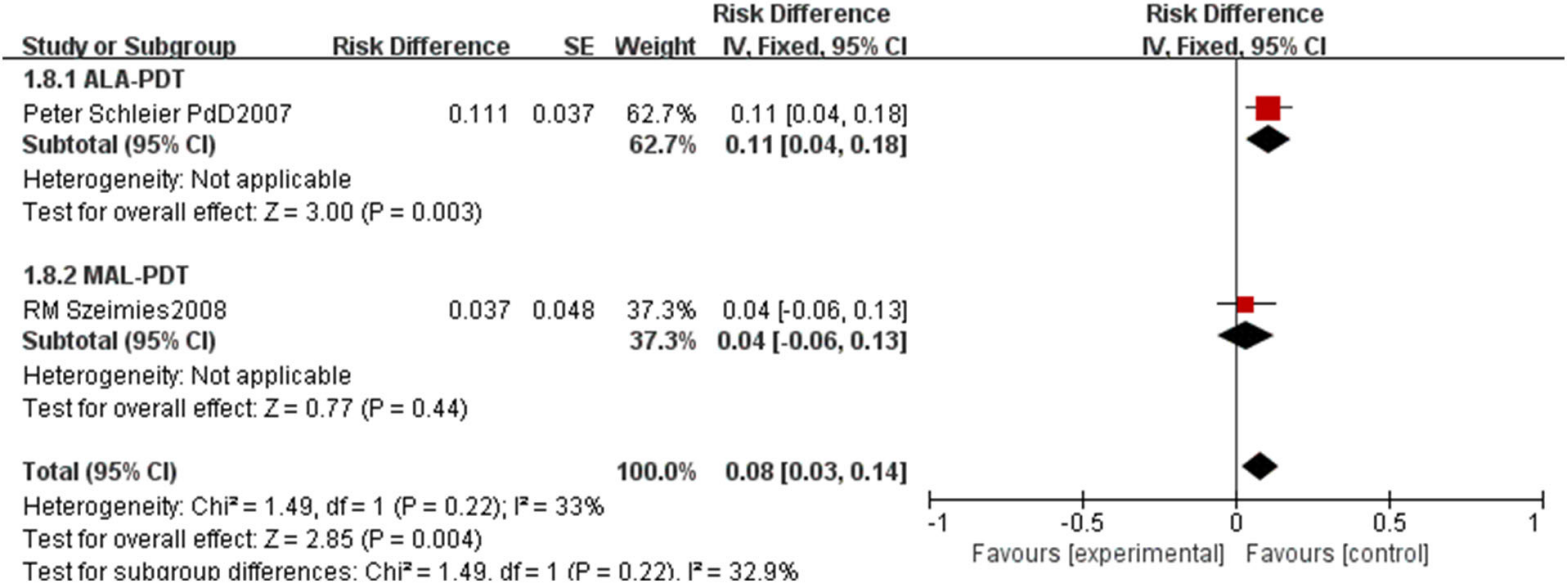
Forest plot of pooled incidence rate of adverse events of ALA/MLA photodynamic therapy for sBCC.

For safety and long-term outcomes, the pooled recurrence rate was 0.13 (95% CI: 0.09–0.18, p<0.00001, **Figure 6**) with high heterogeneity (I²=87%). ALA-PDT had a pooled recurrence rate of 0.12 (95% CI: 0.07–0.18), MAL-PDT of 0.17 (95% CI: 0.07–0.26), and BF-200 ALA-PDT of 0.06 (95% CI: -0.06–0.18), with no significant subgroup differences (p=0.24, I²=0%). The pooled incidence rate of adverse events was 0.08 (95% CI: 0.03–0.14, p=0.004, **Figure 7**) with low heterogeneity (I²=33%), where ALA-PDT subgroup had a higher rate of 0.11 (95% CI: 0.04–0.18) compared to MAL-PDT’s 0.04 (95% CI: -0.06–0.13), though the subgroup difference was not statistically significant (p=0.22, I²=32.9%).

### Nodular basal cell carcinoma

A total of 34 studies focusing on nBCC were included, involving 1136 patients and 1542 lesions. The interventions included ALA-PDT (20%), MAL-PDT, BF-200 ALA-PDT, and AFL-MAL-PDT, with treatment cycles ranging from 1 to 4, light parameters varying in wavelength (417–980 nm), intensity (10–200 mW/cm²), and dose (37–150 J/cm²), and evaluation times spanning 1 month to 5 years. The meta-analysis results demonstrated significant therapeutic effects of PDT for nBCC, with varying degrees of heterogeneity observed across outcome indicators.

**Figure 8:**
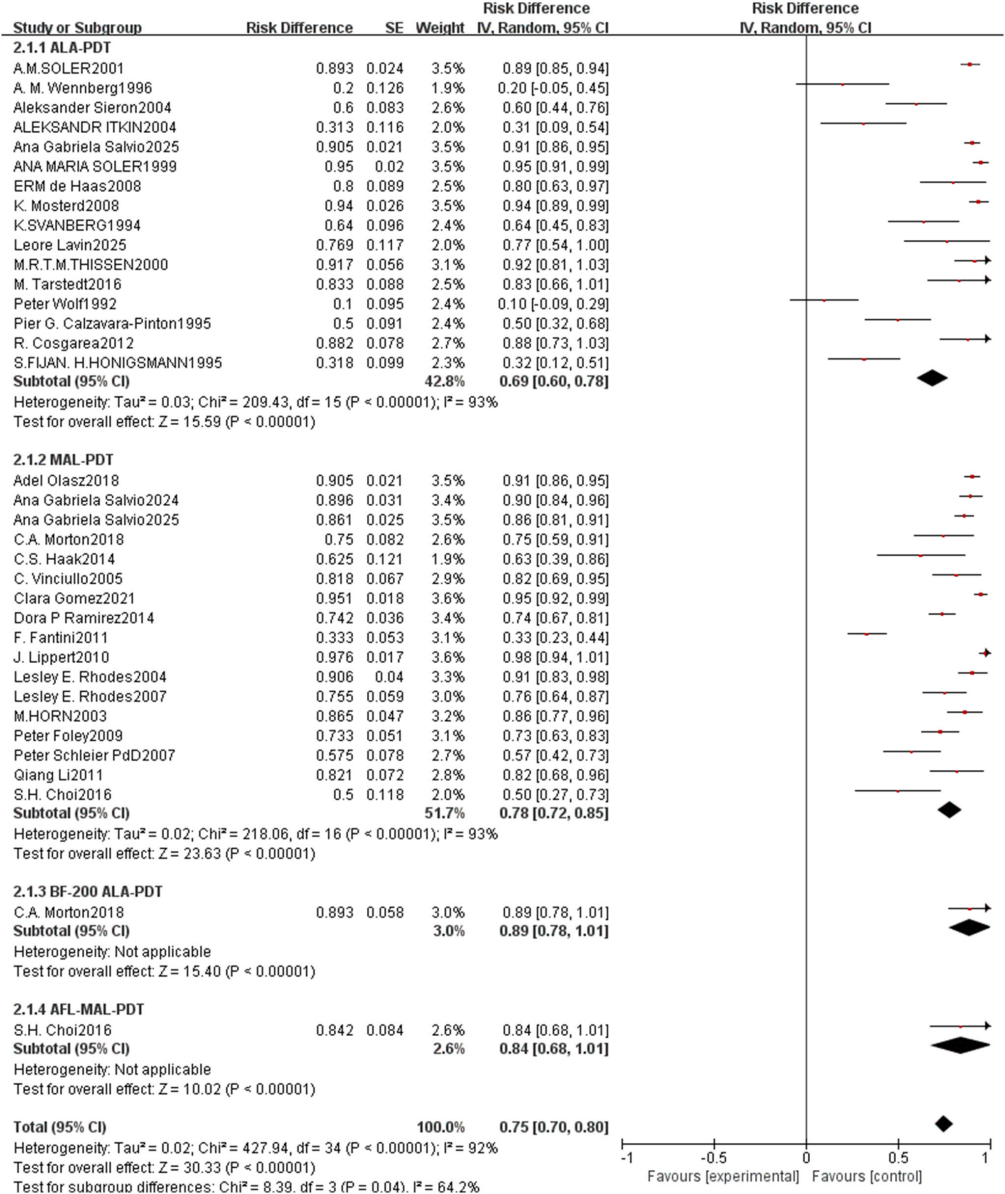
Forest plot of pooled CR rate of ALA/MLA photodynamic therapy for nBCC.

**Figure 9:**
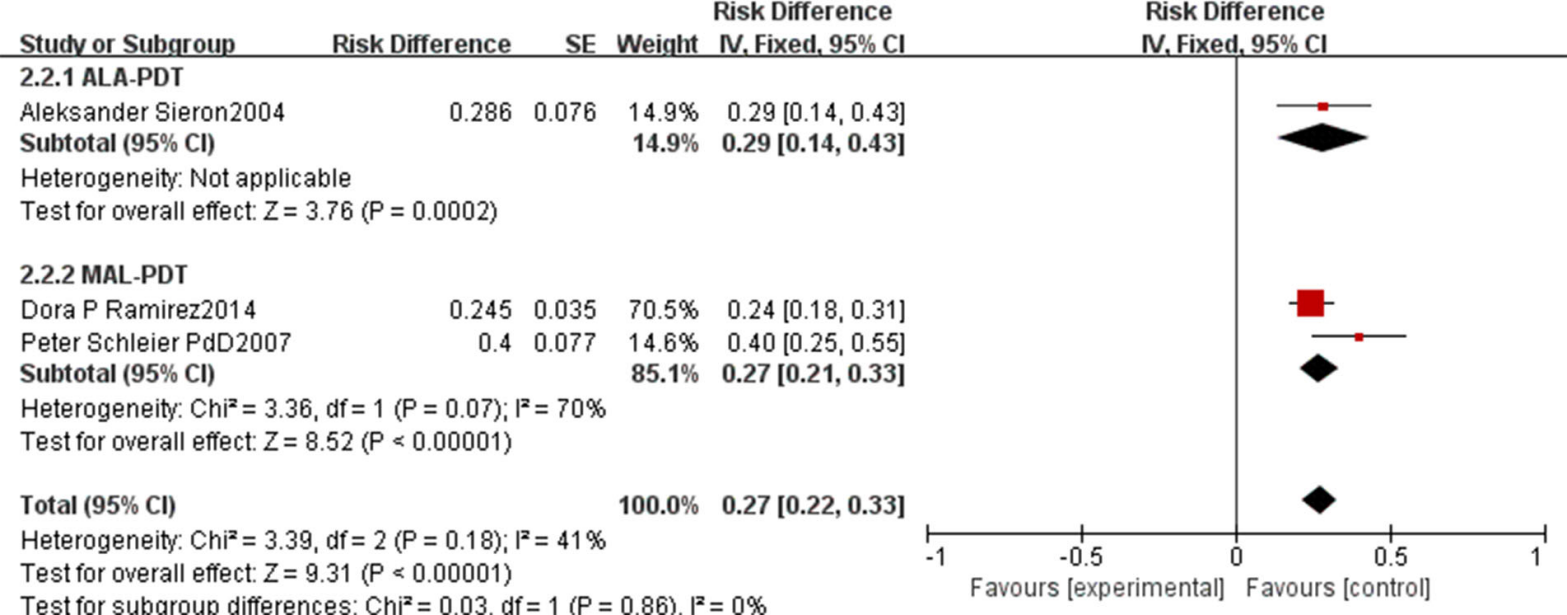
Forest plot of pooled PR rate of ALA/MLA photodynamic therapy for nBCC.

For CR rate, the pooled rate across all included studies was 0.75 (95% CI: 0.70–0.80, p<0.00001, **Figure 8**) with high heterogeneity (I²=92%). Subgroup analysis by PDT type showed that ALA-PDT achieved a pooled CR rate of 0.69 (95% CI: 0.60–0.78), MAL-PDT of 0.78 (95% CI: 0.72–0.85), BF-200 ALA-PDT of 0.89 (95% CI: 0.78–1.01), and AFL-MAL-PDT of 0.84 (95% CI: 0.68–1.01), with a statistically significant difference between subgroups (p=0.04, I²=64.2%). The PR rate had a pooled value of 0.27 (95% CI: 0.22–0.33, p<0.00001, **Figure 9**) with moderate heterogeneity (I²=41%). The ALA-PDT subgroup showed a pooled PR rate of 0.29 (95% CI: 0.14–0.43) and the MAL-PDT subgroup of 0.27 (95% CI: 0.21–0.33), with no significant subgroup difference (p=0.80, I²=70%).

**Figure 10:**
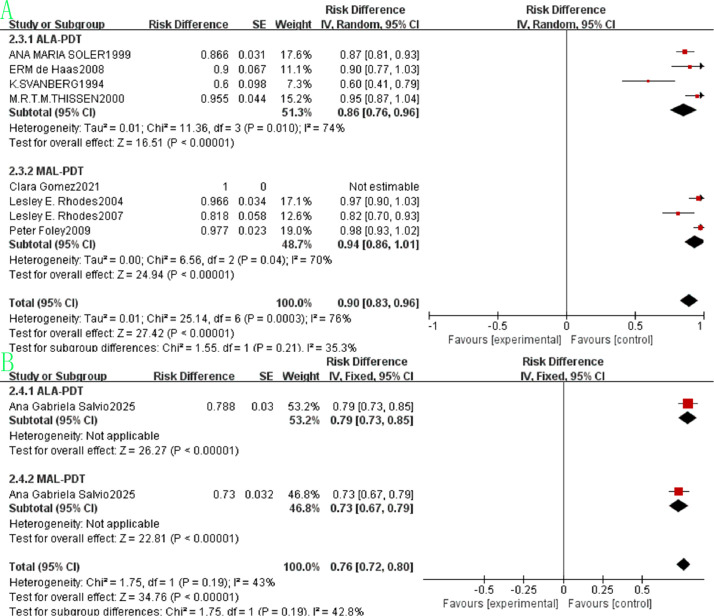
Forest plot of pooled beauty effect rate **(A)** and 5-year survival rate **(B)** of ALA/MLA photodynamic therapy for nBCC.

The pooled beauty effect rate was 0.90 (95% CI: 0.83–0.96, p<0.00001, **Figure 10A**) with substantial heterogeneity (I²=76%). ALA-PDT had a pooled rate of 0.86 (95% CI: 0.76–0.96) and MAL-PDT of 0.94 (95% CI: 0.86–1.01), and the difference between subgroups was not statistically significant (p=0.21, I²=35.3%). Regarding survival outcomes, the pooled 5-year survival rate was 0.76 (95% CI: 0.72–0.80, p<0.00001, **Figure 10B**) with moderate heterogeneity (I²=43%). The ALA-PDT subgroup had a pooled 1-year survival rate of 0.79 (95% CI: 0.73–0.85) and the MAL-PDT subgroup of 0.73 (95% CI: 0.67–0.79), with no significant subgroup difference (p=0.19, I²=42.8%).

**Figure 11:**
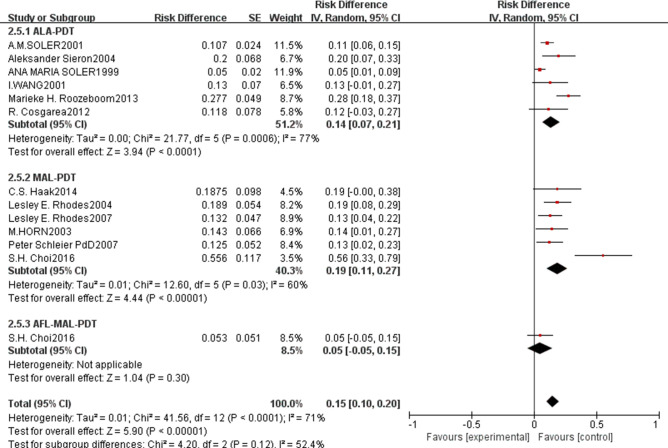
Forest plot of pooled recurrence rate of ALA/MLA photodynamic therapy for nBCC.

**Figure 12:**
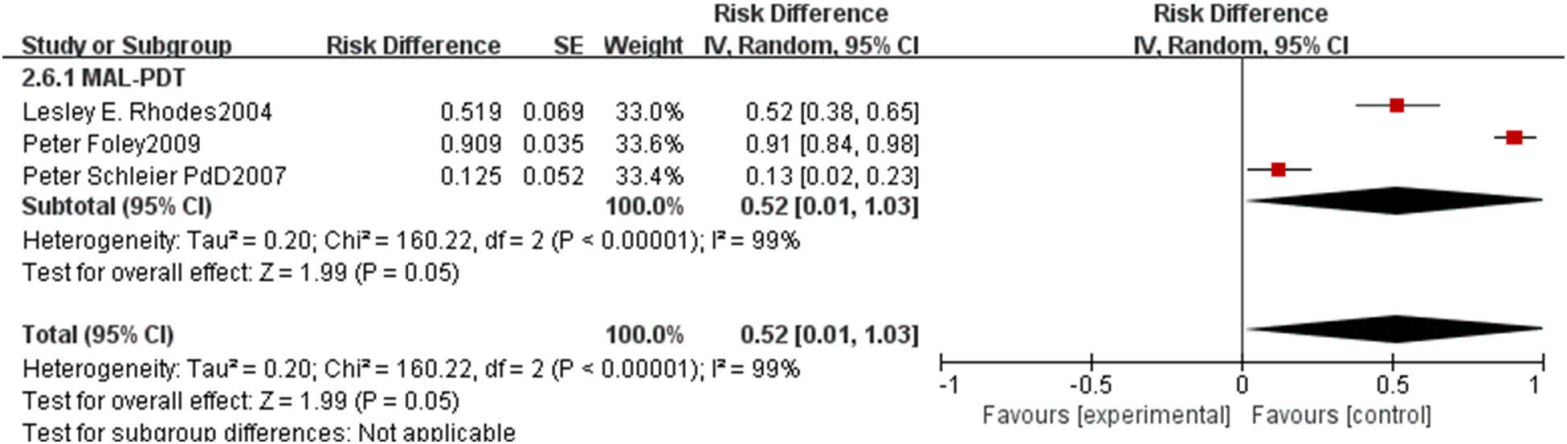
Forest plot of pooled incidence rate of adverse events of ALA/MLA photodynamic therapy for nBCC.

For long-term safety outcomes, the pooled recurrence rate was 0.15 (95% CI: 0.10–0.20, p<0.00001, **Figure 11**) with high heterogeneity (I²=71%). Subgroup analysis revealed that ALA-PDT had a pooled recurrence rate of 0.14 (95% CI: 0.07–0.21), MAL-PDT of 0.19 (95% CI: 0.11–0.27), and AFL-MAL-PDT of 0.05 (95% CI: -0.05–0.15), with no statistically significant difference between subgroups (p=0.12, I²=52.4%). Due to limited available data, the incidence rate of adverse events for nBCC could not be pooled for comprehensive analysis(**Figure 12**), and individual study reports indicated mild to moderate local reactions that were manageable with supportive care.

### Subgroup analysis for heterogeneity

To explore the sources of high heterogeneity observed in key outcomes of sBCC, subgroup analyses were performed based on study design, light dose, number of treatments, and follow-up period (**Supplementary pictures**). For CR rate (I²=87%), stratified analysis by study design showed pooled rates of 0.91 (95% CI: 0.89–0.93, I²=75%) in real-world studies and 0.83 (95% CI: 0.77–0.89, I²=90%) in RCTs, with a significant subgroup difference (p=0.01). Further analysis revealed that light dose (37–80 J/cm²) and ≥3 treatment sessions were associated with higher CR rates (0.91 vs. 0.82, p=0.01; 0.92 vs. 0.85, p=0.02, respectively). For other outcomes, the beauty effect rate and recurrence rate also showed reduced heterogeneity when stratified by light dose and follow-up duration, indicating that standardized treatment parameters and prolonged follow-up may improve result consistency.

For nBCC, similar subgroup analyses were conducted to address high heterogeneity in CR rate (I²=92%) and other outcomes. Stratification by study design yielded pooled CR rates of 0.71 (95% CI: 0.64–0.78, I²=95%) in real-world studies and 0.83 (95% CI: 0.77–0.88, I²=73%), with a statistically significant difference (p=0.01). Light dose >50 J/cm² was associated with a higher CR rate (0.83 vs. 0.78, p=0.05), while ≥2 treatment sessions significantly improved efficacy compared to single sessions (0.80 vs. 0.68, p=0.04). For recurrence rate (I²=71%), subgroup analysis by treatment sessions and follow-up period showed reduced heterogeneity, suggesting that optimized treatment frequency and long-term monitoring can mitigate variability in long-term outcomes. These findings highlight that standardizing key treatment parameters and distinguishing study design types are critical for reducing heterogeneity in PDT efficacy evaluations for BCC.

## Discussion

This meta-analysis comprehensively evaluated the efficacy and safety of ALA-PDT and MAL-PDT in the treatment of superficial and nodular basal cell carcinoma, synthesizing data from 55 studies involving over 2000 patients and 3000 lesions. The key findings highlight that PDT achieves significant therapeutic effects across both BCC subtypes, with notable differences in response rates between sBCC and nBCC, and varying performance among different photosensitizer formulations. These results align with and extend previous clinical evidence, while addressing critical gaps in our understanding of PDT’s role in BCC management.

The pooled CR rate for sBCC (0.88, 95% CI: 0.85–0.91) was substantially higher than that for nBCC (0.75, 95% CI: 0.70–0.80), consistent with the biological characteristics of the two subtypes. Superficial BCCs are confined to the papillary dermis, allowing better penetration of topical photosensitizers and more effective light activation, whereas nodular BCCs exhibit deeper invasion into the reticular dermis and often thicker tumor nests, limiting PDT’s cytotoxic effects (10, 26). Rhodes et al. (4) previously reported a 91% CR rate for MAL-PDT in nBCC at 3 months, but a higher recurrence rate (17%) at 12 months compared to surgery, which is supported by our finding of a pooled recurrence rate of 0.15 for nBCC treated with PDT. This suggests that while PDT is effective for nBCC, its long-term disease control may be inferior to surgical excision, particularly for thicker lesions (>2 mm), as also noted in the European Dermatology Forum guidelines (9).

Subgroup analysis by photosensitizer type revealed that MAL-PDT achieved a higher CR rate than ALA-PDT in both sBCC (0.90 vs. 0.87) and nBCC (0.78 vs. 0.69), with a statistically significant difference among nBCC subgroups (p=0.04). This may be attributed to MAL’s enhanced lipophilicity, which improves skin penetration and protoporphyrin IX (PpIX) accumulation in deeper tumor tissue compared to ALA (67, 68). Additionally, novel formulations such as BF-200 ALA (a nanoemulsion-based gel) showed promising results, with a CR rate of 0.90 for sBCC and 0.89 for nBCC, likely due to improved solubility and targeted delivery (53). Morton et al. (53) conducted a phase III trial demonstrating non-inferiority of BF-200 ALA to MAL in non-aggressive BCC, which is consistent with our subgroup findings and supports the potential of optimized formulations to enhance PDT efficacy.

The beauty effect rate, a critical outcome for patient satisfaction, was consistently high across both subtypes (sBCC: 0.91; nBCC: 0.90), with no significant difference between ALA-PDT and MAL-PDT. This aligns with previous studies highlighting PDT’s superior cosmetic outcomes compared to surgery, as it avoids tissue excision and minimizes scarring. Soler et al. (29) reported that 89% of patients treated with MAL-PDT for BCC rated their cosmetic outcome as excellent or good at 3 years, which is comparable to our pooled results and reinforces PDT’s advantage in preserving skin aesthetics, especially in visible areas.

Regarding safety, the pooled incidence rate of adverse events was low (sBCC: 0.08; nBCC: data insufficient for pooling), with mild to moderate local reactions (erythema, pain, edema) being the most common. These reactions are transient and manageable, consistent with the safety profile of PDT reported in other meta-analyses. Nestor et al. (69) noted that ALA-PDT-related adverse events resolved within 1–2 weeks without long-term sequelae, supporting PDT’s tolerability for elderly or frail patients who may not tolerate surgery.

Numerous systematic reviews and meta-analyses have explored the application of PDT in BCC treatment (14–16), and the present study supplements and advances the existing research in multiple dimensions with distinctive design and analysis depth. In terms of research timeliness and inclusion scope, this study incorporates clinical studies published up to December 31, 2025, with a total of 55 studies included in the meta-analysis, which is a larger sample size compared with most previous relevant studies. More importantly, this research enriches the research evidence by including novel PDT photosensitizer formulations such as BF-200 ALA and AFL-MAL, which were only partially reported or not fully analyzed in earlier reviews. In terms of study design stratification, previous studies mostly adopted a single combined analysis of RCTs and observational studies, while this study subdivides the included research into randomized controlled trials and real-world studies (prospective and retrospective cohort studies), and conducts stratified efficacy analysis for different design types, making the research results more targeted for clinical reference. In terms of therapeutic parameter analysis, existing studies only briefly mentioned the heterogeneity of PDT treatment parameters such as light wavelength and dose among different studies, but lacked in-depth quantitative analysis of the correlation between these parameters and treatment efficacy. This study fills this gap by systematically sorting out and analyzing key treatment parameters. In addition, in the exploration of heterogeneity, previous studies mostly only carried out simple subgroup analysis based on PDT type or BCC subtype, while this study further expanded the subgroup analysis dimensions to quantify the contribution of different factors to heterogeneity, making the heterogeneity exploration more comprehensive and in-depth.

This meta-analysis provides evidence-based clinical guidance for the application of ALA/MAL-PDT in the treatment of sBCC and nBCC, clarifying the clinical positioning, optimal application strategies and key technical parameters of PDT in BCC management. First, in terms of treatment selection, ALA/MAL-PDT has demonstrated high CR rates, excellent cosmetic outcomes and a favorable safety profile in both sBCC and nBCC, with only mild and transient local adverse events such as erythema and pain. This confirms that PDT is a first-line non-invasive treatment option for sBCC and nBCC patients, especially for those with lesions in cosmetically sensitive areas (e.g., face, neck), patients with multiple BCC lesions, and elderly or frail patients with contraindications to surgical excision or poor surgical tolerance. Second, the study identifies the key optimized treatment parameters for PDT in BCC through in-depth subgroup analysis: a light dose of 37–80 J/cm² and at least 3 treatment sessions are associated with higher CR rates for sBCC, while a light dose >50 J/cm² and ≥2 treatment sessions can significantly improve the therapeutic effect of nBCC. These findings provide clear and operable technical standards for clinical PDT implementation, helping to standardize treatment operations and improve the consistency of therapeutic effects. Third, in the selection of photosensitizers, MAL-PDT is recommended as the first choice for nBCC due to its statistically significant advantage in CR rate, which is attributed to MAL’s strong lipophilicity and better penetration in deep nodular tumor tissue. For sBCC, clinicians can flexibly select ALA-PDT or MAL-PDT according to patient’s economic status, local drug availability and individual tolerance. In addition, novel formulations such as BF-200 ALA-PDT show high CR rates in both sBCC and nBCC, which is a promising new direction for PDT in BCC treatment, and is worthy of further clinical promotion and large-sample long-term follow-up research. Fourth, for the high heterogeneity observed in PDT efficacy evaluation, clinical practice should pay attention to standardizing treatment protocols and recording detailed clinical data (e.g., lesion thickness, light source parameters, follow-up duration), which is conducive to the accumulation of high-quality real-world evidence and the continuous optimization of PDT treatment strategies for BCC.

Several limitations of this meta-analysis should be acknowledged. First, high heterogeneity was observed across most outcome indicators (e.g., sBCC CR rate: I²=87%; nBCC CR rate: I²=92%), which may be attributed to variations in treatment parameters and study design (single-arm vs. RCT) (70). Second, some studies lacked detailed data on lesion thickness, a key determinant of PDT response, which may have influenced subgroup analyses. Third, long-term follow-up data (≥5 years) were limited for certain subgroups, particularly for novel formulations like BF-200 ALA and AFL-MAL, making it difficult to assess long-term recurrence risk. Finally, publication bias cannot be excluded, as smaller studies with negative results may be underreported.

Future research directions should focus on the following aspects: 1) Standardizing PDT treatment protocols: Conduct multicenter RCTs to verify the optimal treatment parameters summarized in this study and formulate unified clinical guidelines to reduce heterogeneity across studies. 2) Comparing ALA-PDT and MAL-PDT in matched cohorts: Conduct RCTs directly comparing ALA-PDT and MAL-PDT in matched sBCC and nBCC cohorts to confirm subgroup differences observed in this meta-analysis. 3) Exploring combination therapies: Investigate combination therapies (e.g., PDT combined with curettage, targeted drugs) to enhance efficacy for thicker or aggressive BCC subtypes. 4) Long-term follow-up of novel formulations: Conduct long-term prospective studies to evaluate the durability of response and cosmetic outcomes for novel formulations such as BF-200 ALA and AFL-MAL.

In conclusion, ALA/MAL-PDT is a clinically valuable non-invasive treatment for sBCC and nBCC, characterized by high efficacy, excellent cosmetic outcomes and good safety, and it can serve as an important alternative to surgical excision in clinical BCC management. MAL-PDT has a clear efficacy advantage in the treatment of nBCC, while ALA-PDT and MAL-PDT are equally effective for sBCC, and novel PDT formulations represented by BF-200 ALA-PDT show great application potential and are expected to further improve the therapeutic effect of PDT for BCC. The high heterogeneity in the current PDT efficacy research is mainly related to the non-standardization of treatment parameters and the differences in study design, and the standardization of key treatment parameters (e.g., light dose, treatment sessions) is the key to improving the consistency of PDT therapeutic effects. Although PDT has shown good clinical performance, its long-term disease control effect for thicker nBCC lesions is still inferior to surgical excision, and careful patient selection and individualized treatment protocol formulation are essential in clinical application. This study systematically summarizes the efficacy and safety of ALA/MAL-PDT for different BCC subtypes and clarifies the optimal treatment parameters, providing important evidence-based guidance for clinical decision-making. Future research should focus on multicenter randomized controlled trials and standardized real-world research to further verify the long-term efficacy of novel PDT formulations, explore the optimal combination therapy for aggressive BCC subtypes, and continuously improve the clinical application system of PDT in BCC treatment.

## Conclusions

ALA/MAL-PDT is an effective and safe non-invasive therapeutic option for both sBCC and nBCC, with excellent cosmetic outcomes for both subtypes. MAL-PDT exhibits significantly superior efficacy in nBCC compared with ALA-PDT, while the two photosensitizers show comparable therapeutic effects in sBCC. Novel PDT formulations including BF-200 ALA-PDT and AFL-MAL-PDT demonstrate promising CR rates for BCC, providing new treatment alternatives for clinical practice. Standardization of treatment parameters (e.g., light dose, treatment sessions) and differentiation of study design types can effectively reduce heterogeneity in PDT efficacy evaluation, and ALA/MAL-PDT should be prioritized for patients seeking minimally invasive treatment, those with multiple lesions, or those with contraindications to surgical excision.

## Data Availability

The original contributions presented in the study are included in the article/**Supplementary Material**. Further inquiries can be directed to the corresponding author.
