# Supplementary material for: Efficacy and safety of ALA/MLA photodynamic therapy for superficial and nodular basal cell carcinoma: a systematic review and meta-analysis
Source: Front Oncol. 2026 Apr 14;16:1802984. doi: 10.3389/fonc.2026.1802984 (PMC13121321; doi:10.3389/fonc.2026.1802984)
Supplement: Supplementary file 1 [file DataSheet1.docx]

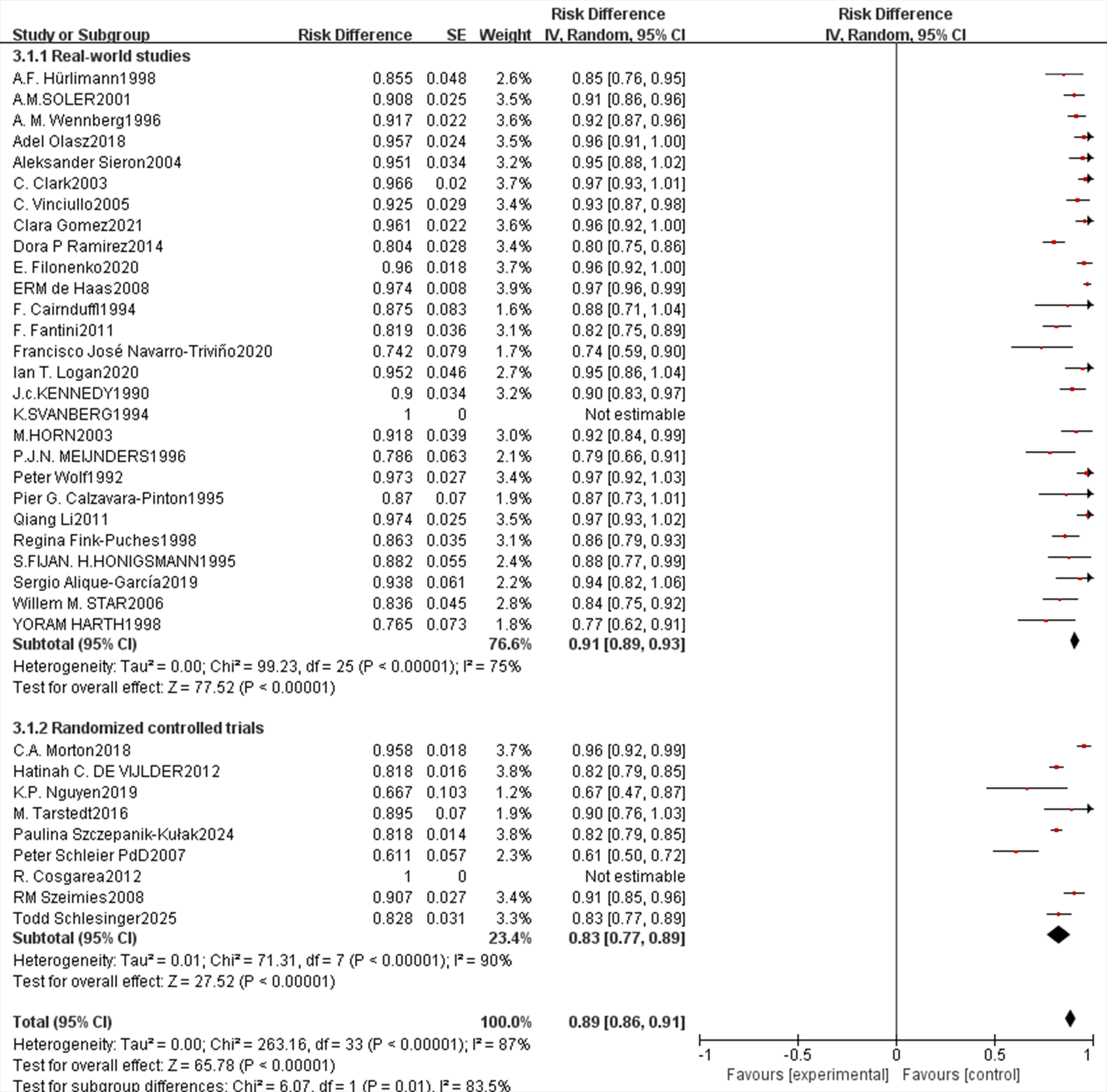


Supplementary Picture 1 Forest plot of CR rate for superficial basal cell carcinoma stratified by study design.


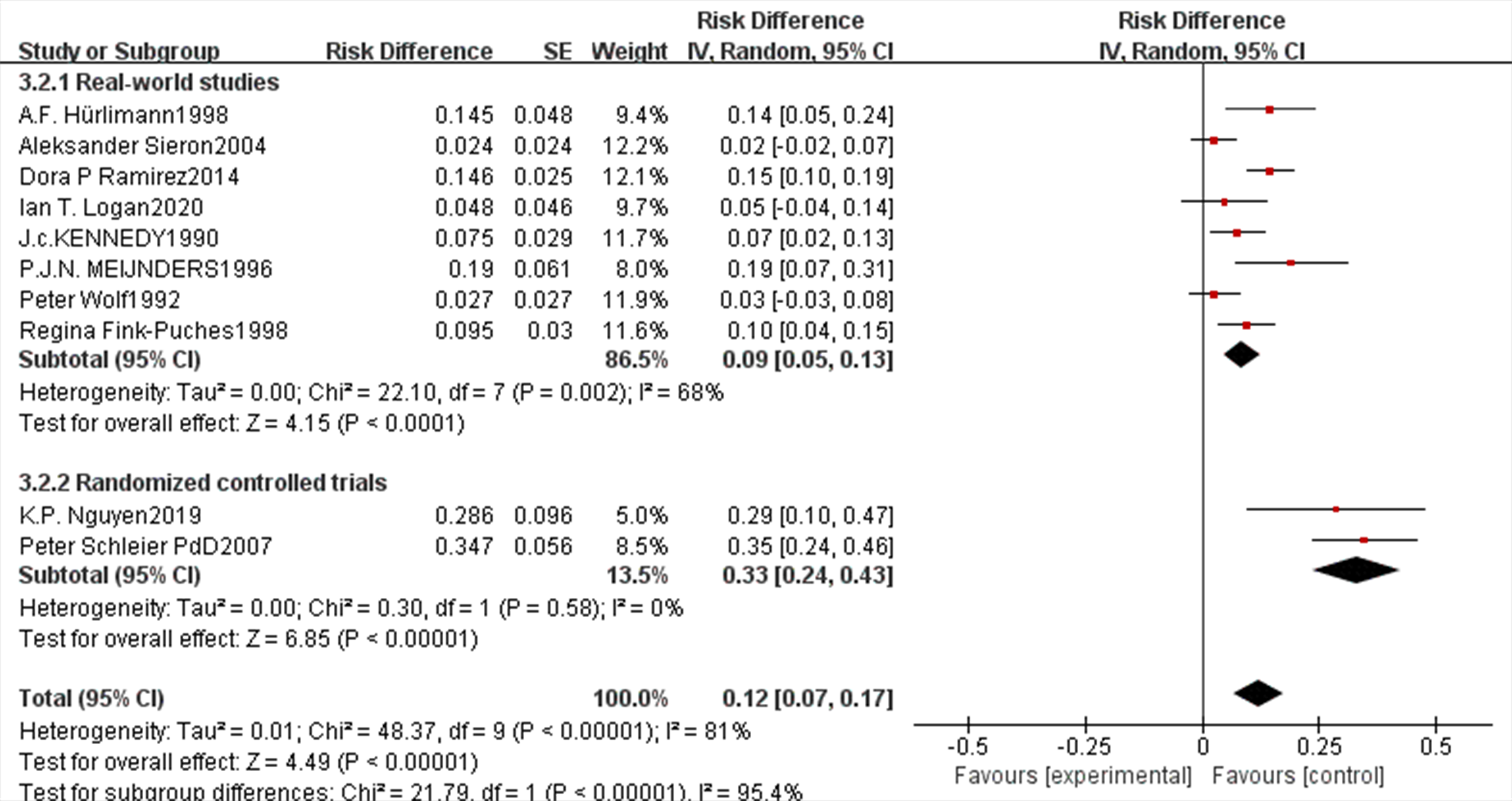


Supplementary Picture 2 Forest plot of PR rate for superficial basal cell carcinoma stratified by study design.


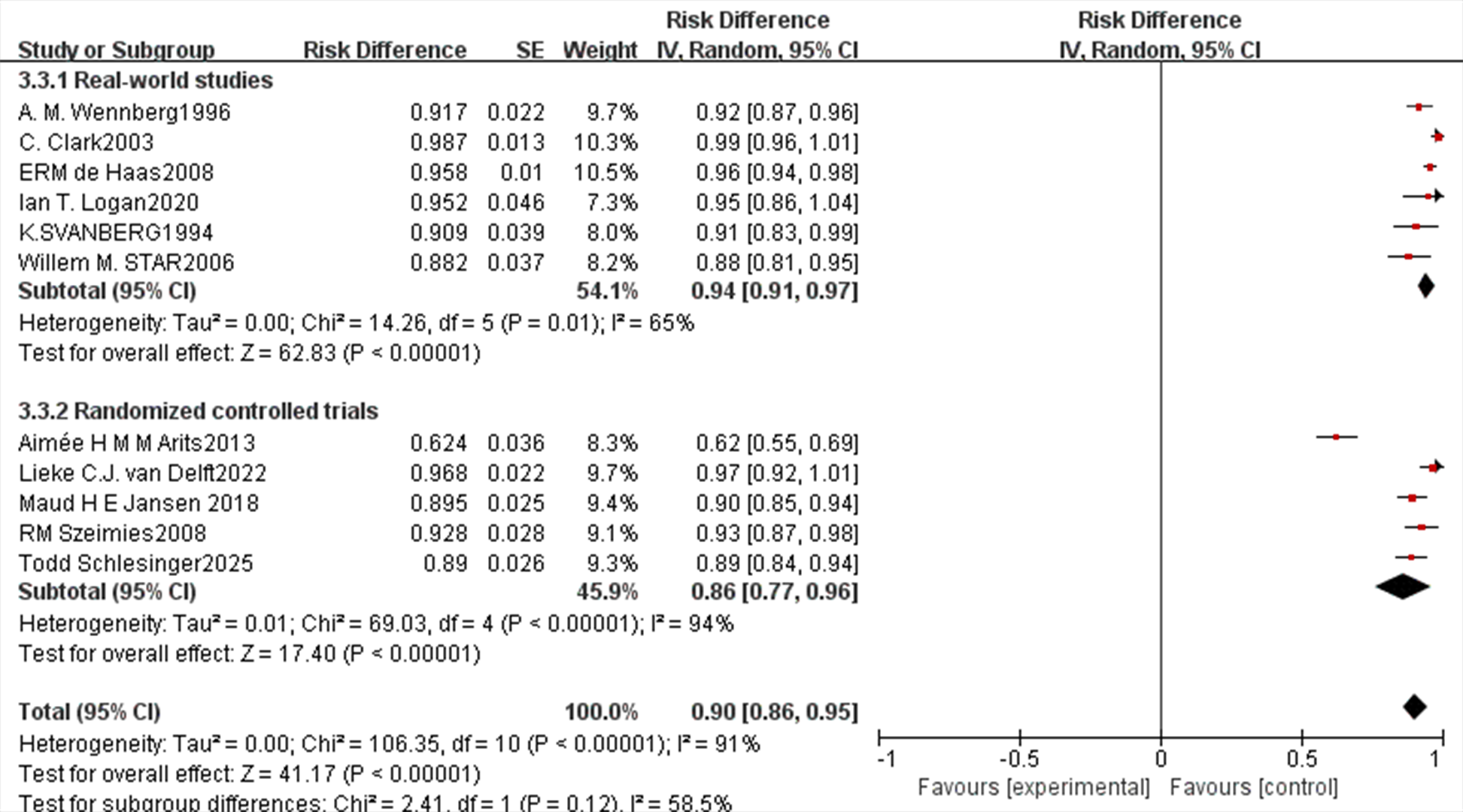


Supplementary Picture 3 Forest plot of beauty effect rate for superficial basal cell carcinoma stratified by study design.


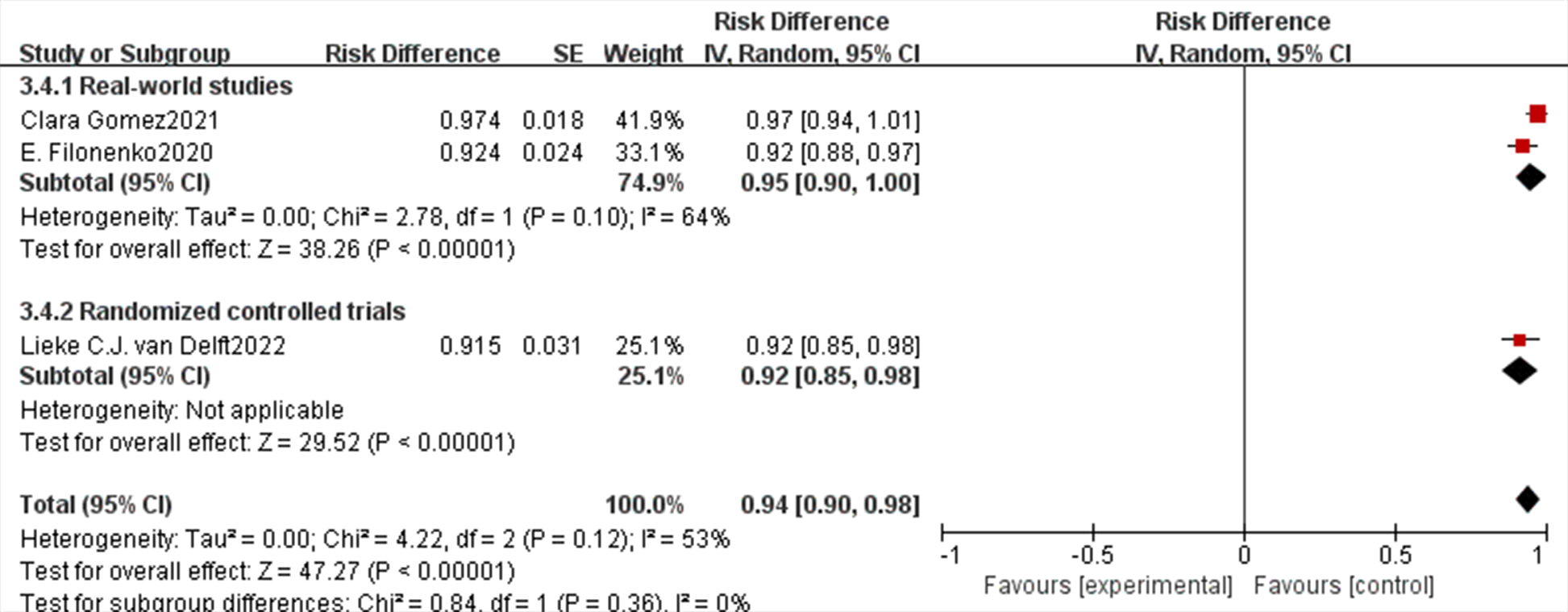


Supplementary Picture 4 Forest plot of One-year survival rate for superficial basal cell carcinoma stratified by study design.


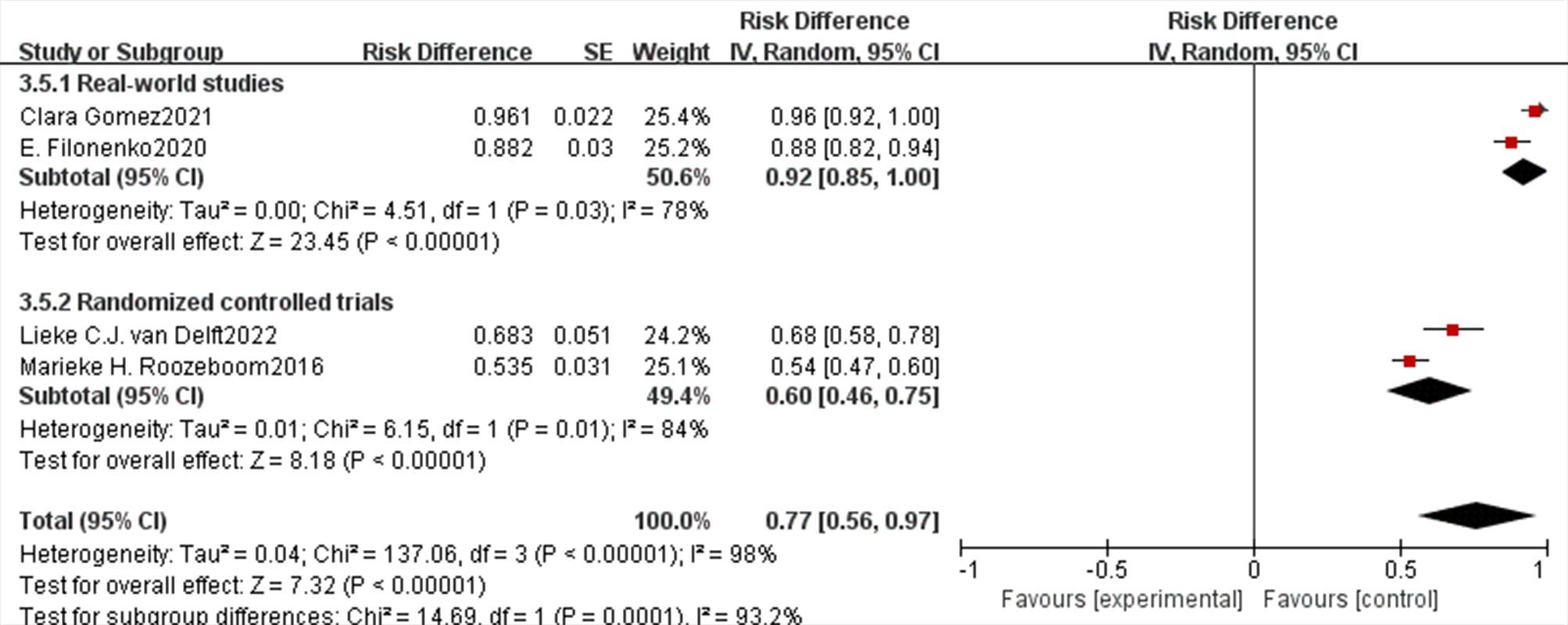


Supplementary Picture 5 Forest plot of Three-year survival rate for superficial basal cell carcinoma stratified by study design.


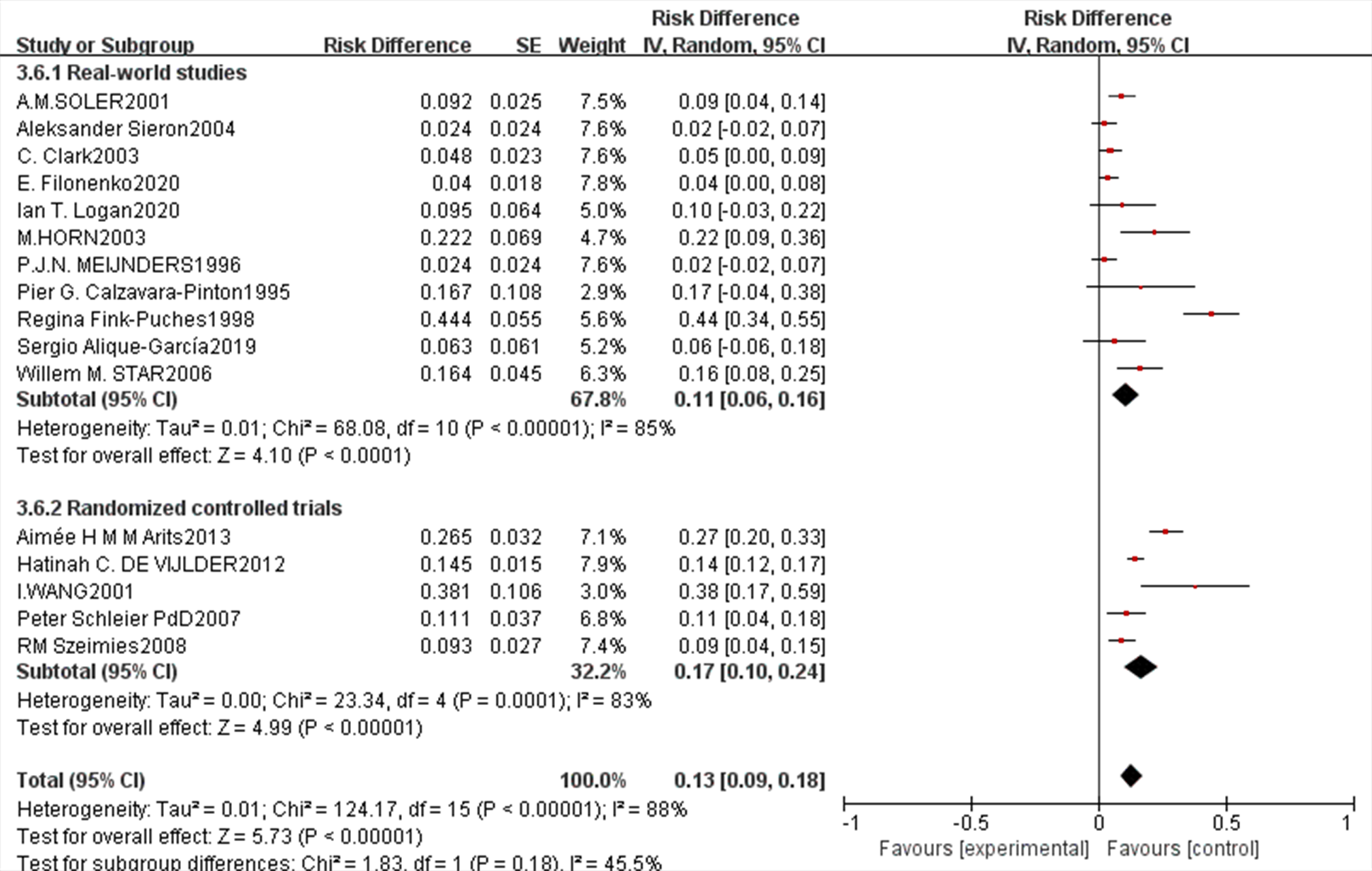


Supplementary Picture 6 Forest plot of Recurrent probability for superficial basal cell carcinoma stratified by study design.


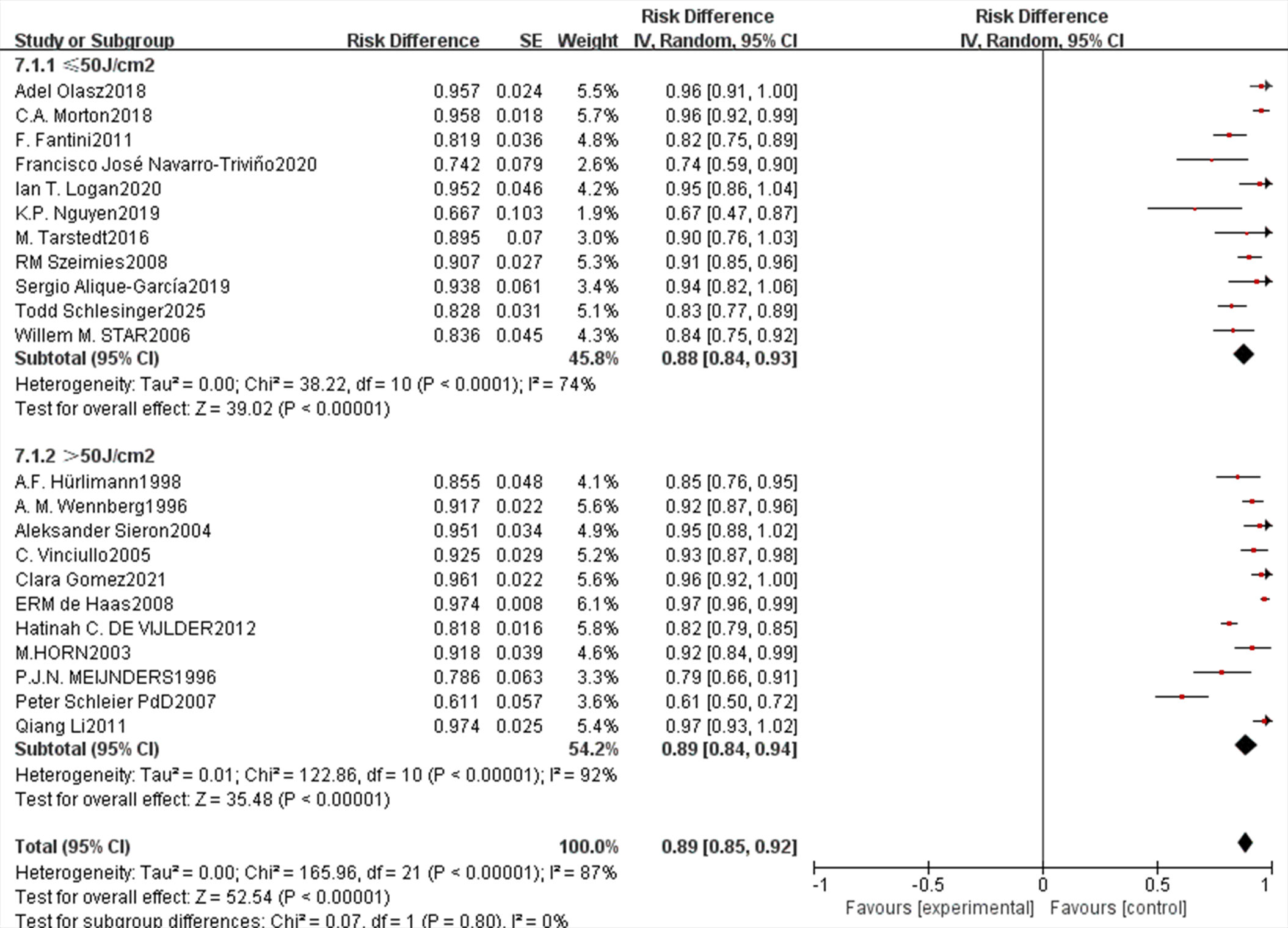


Supplementary Picture 7 Forest plot of CR rate for superficial basal cell carcinoma stratified by Light dose.


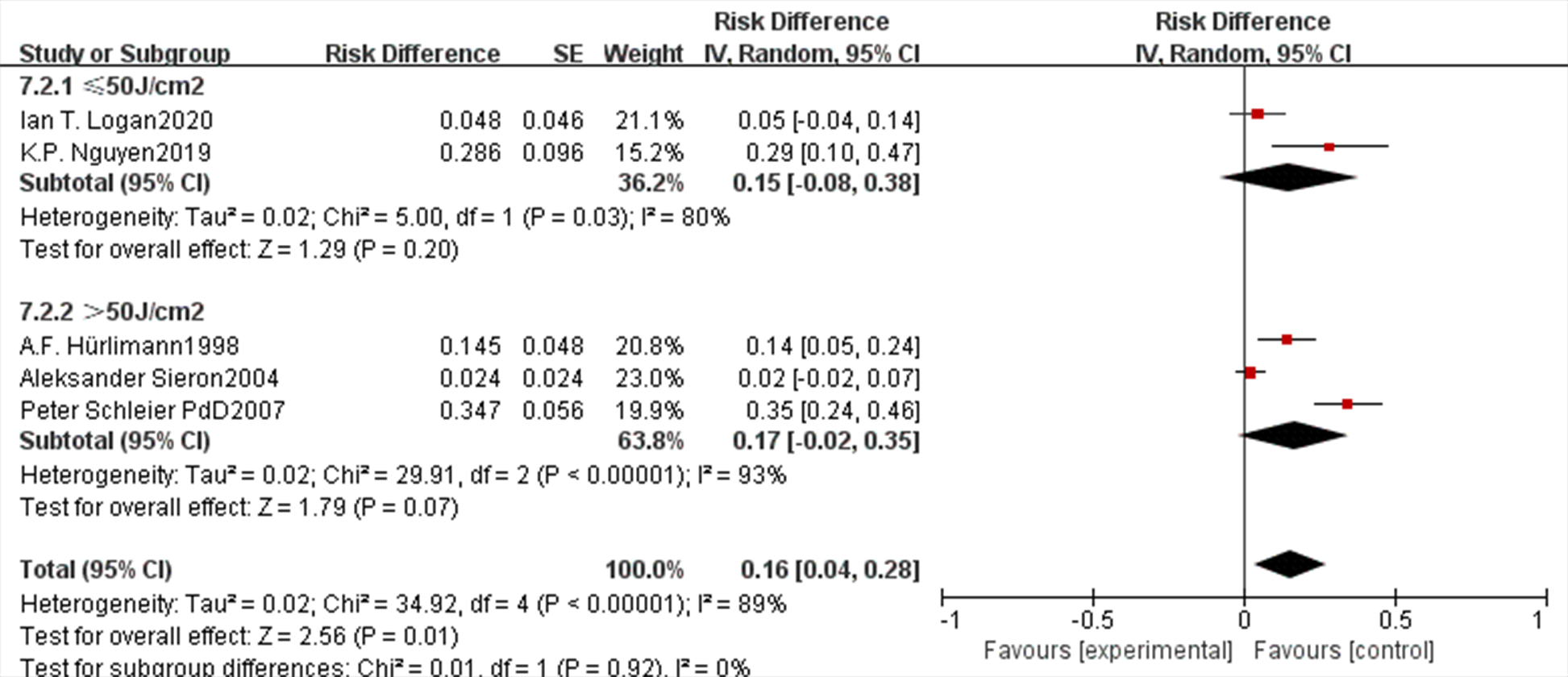


Supplementary Picture 8 Forest plot of PR rate for superficial basal cell carcinoma stratified by Light dose.


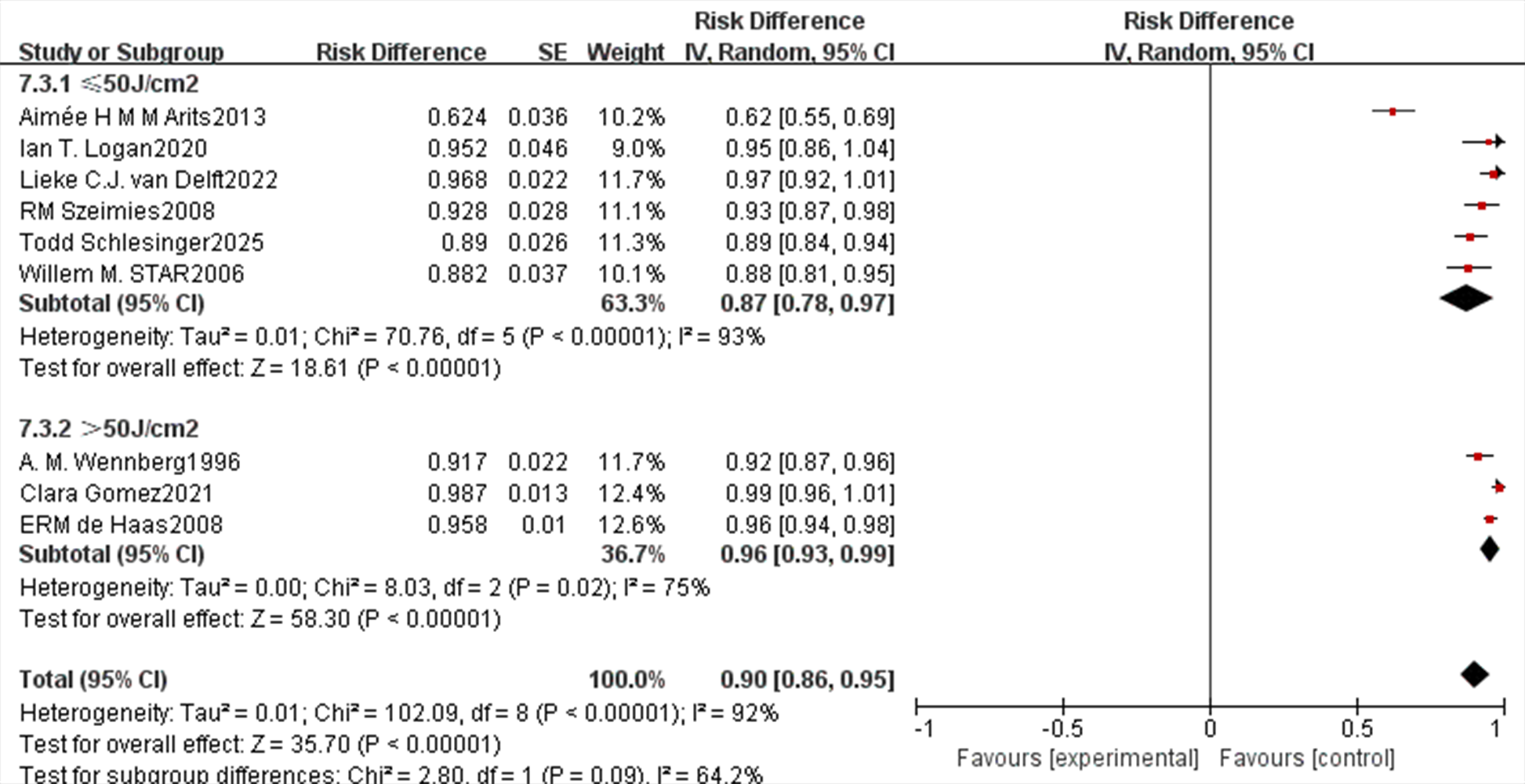


Supplementary Picture 9 Forest plot of Beauty effect rate for superficial basal cell carcinoma stratified by Light dose.


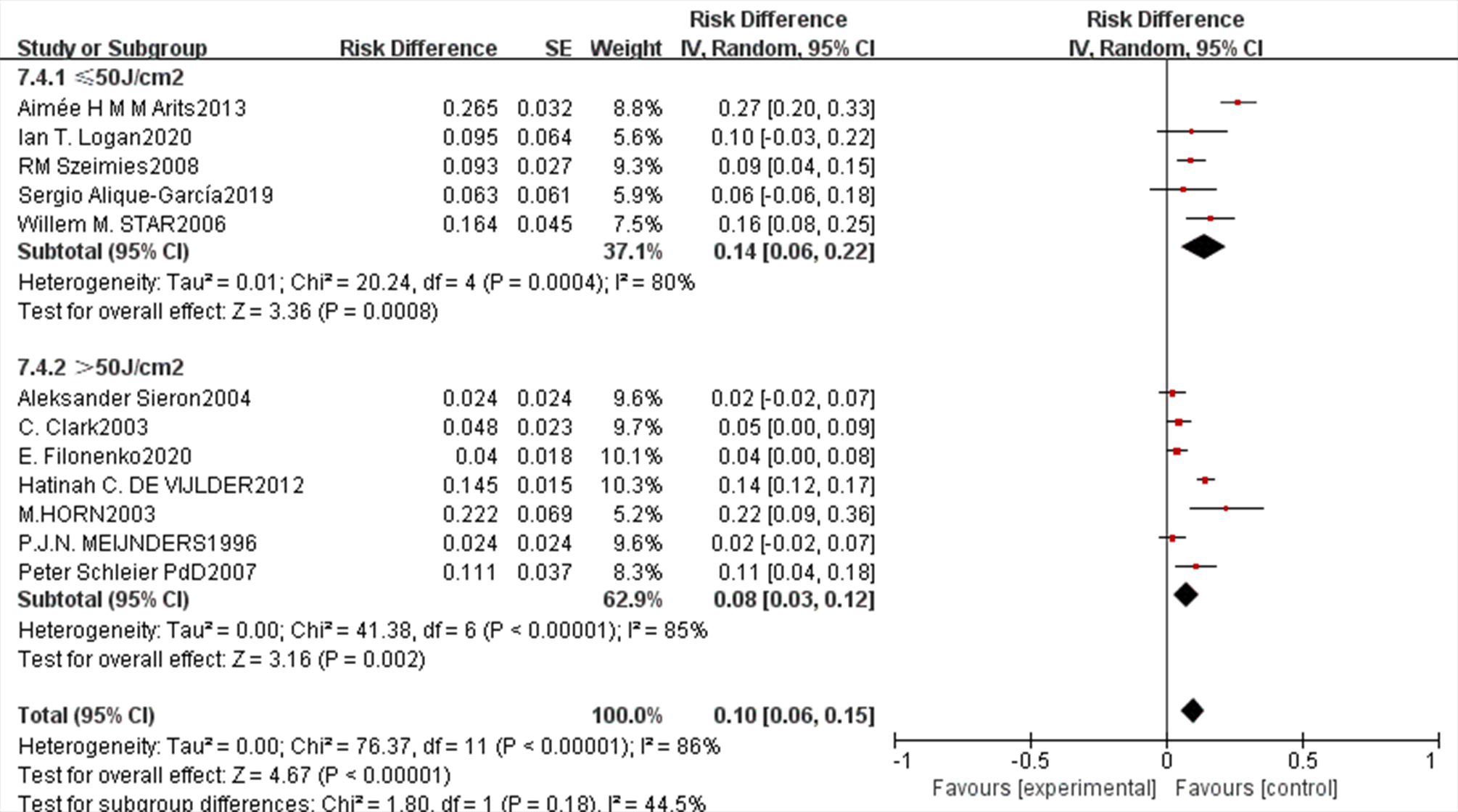


Supplementary Picture 10 Forest plot of Recurrent probability for superficial basal cell carcinoma stratified by Light dose.


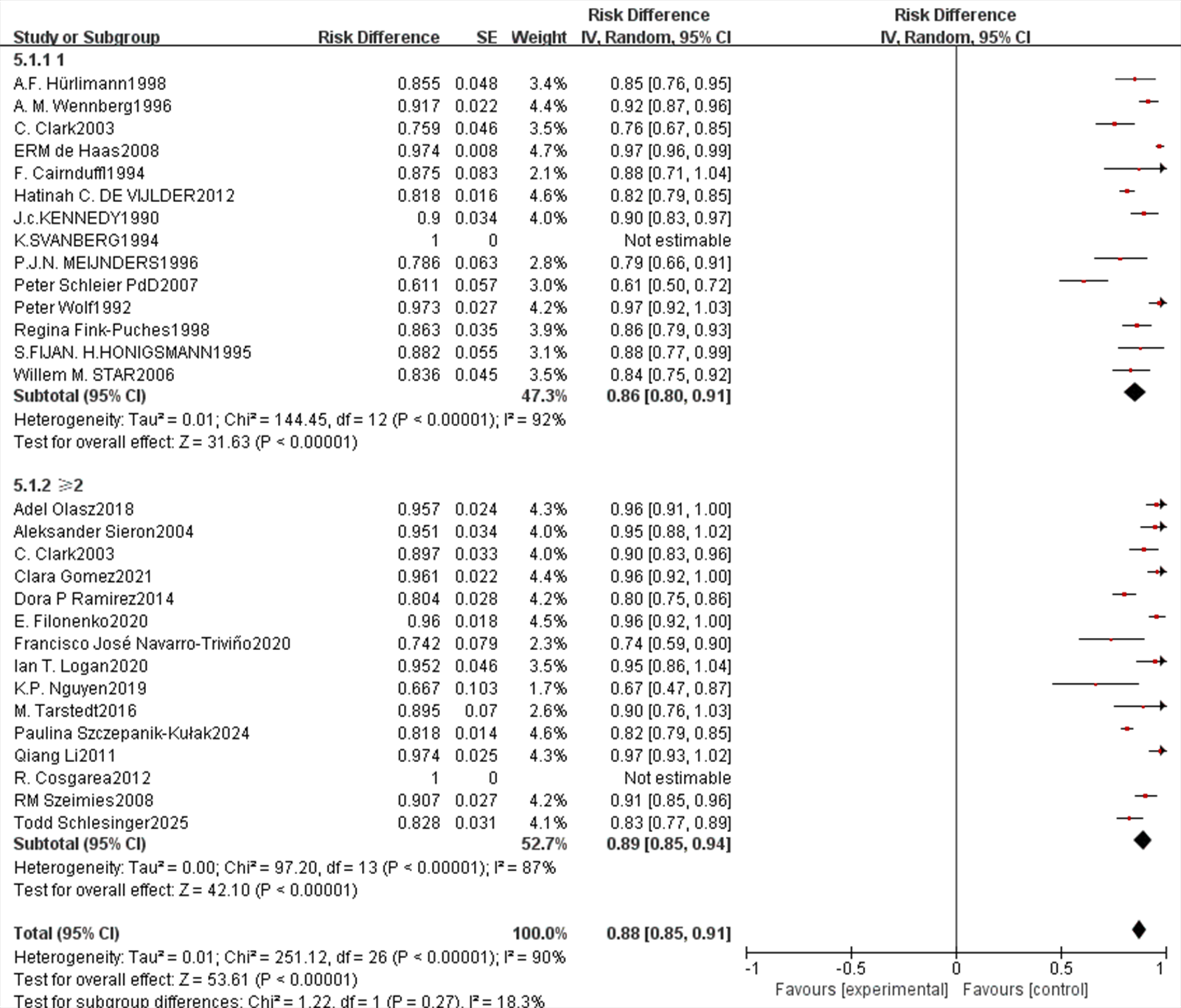


Supplementary Picture 11 Forest plot of CR rate for superficial basal cell carcinoma stratified by Number of treatments.


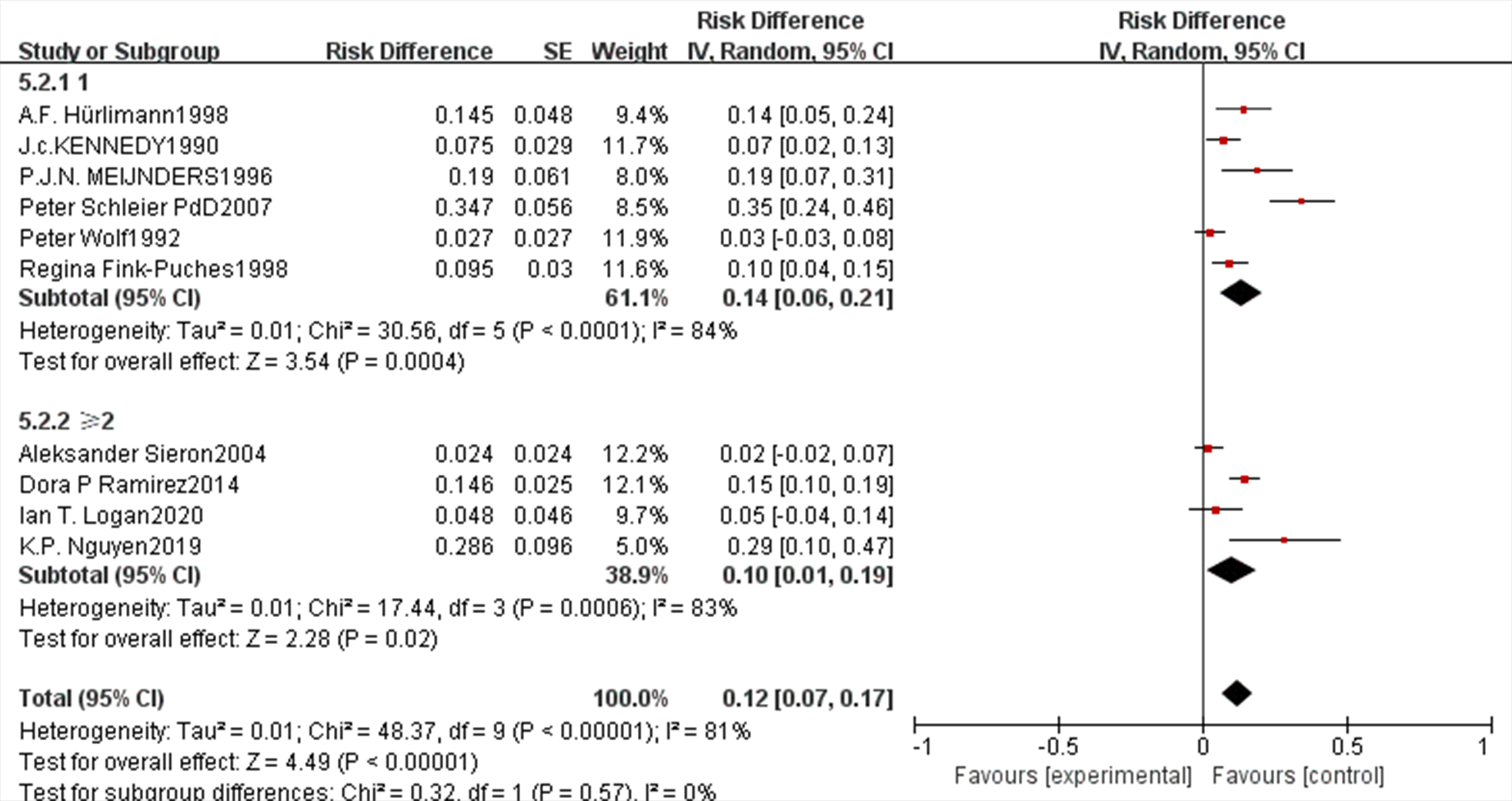


Supplementary Picture 12 Forest plot of PR rate for superficial basal cell carcinoma stratified by Number of treatments.


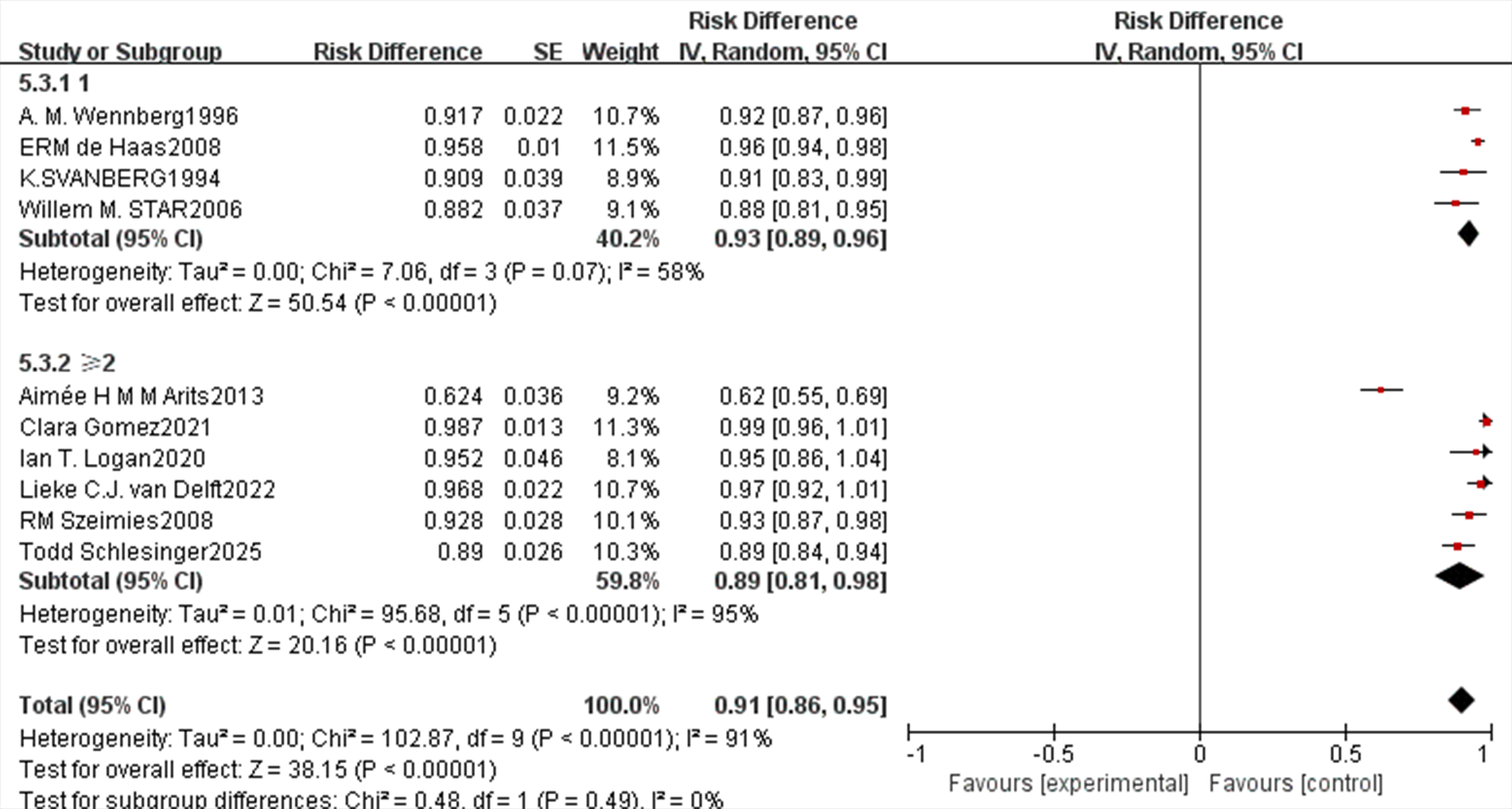


Supplementary Picture 13 Forest plot of Beauty effect rate for superficial basal cell carcinoma stratified by Number of treatments.


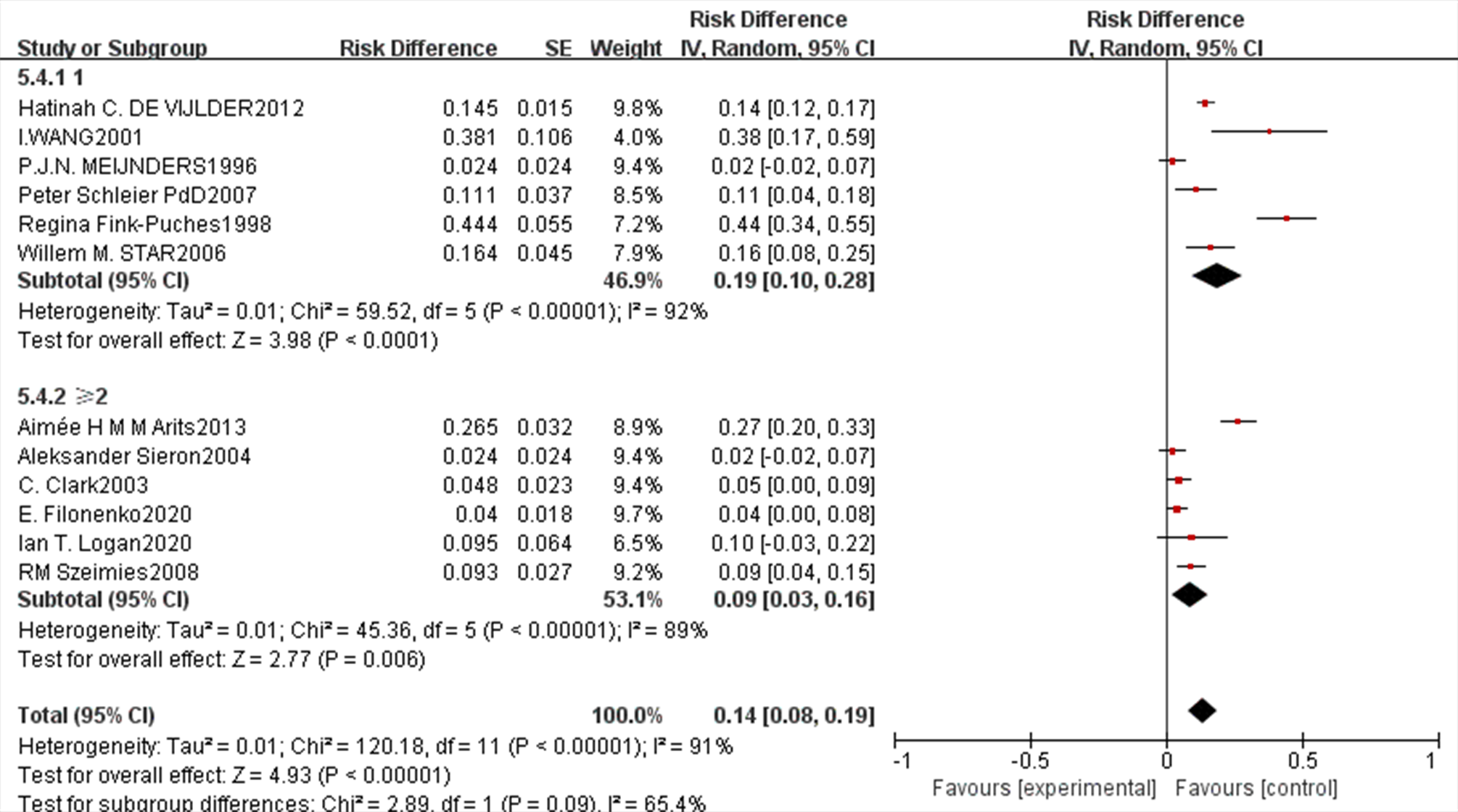


Supplementary Picture 14 Forest plot of Recurrent probability for superficial basal cell carcinoma stratified by Number of treatments.


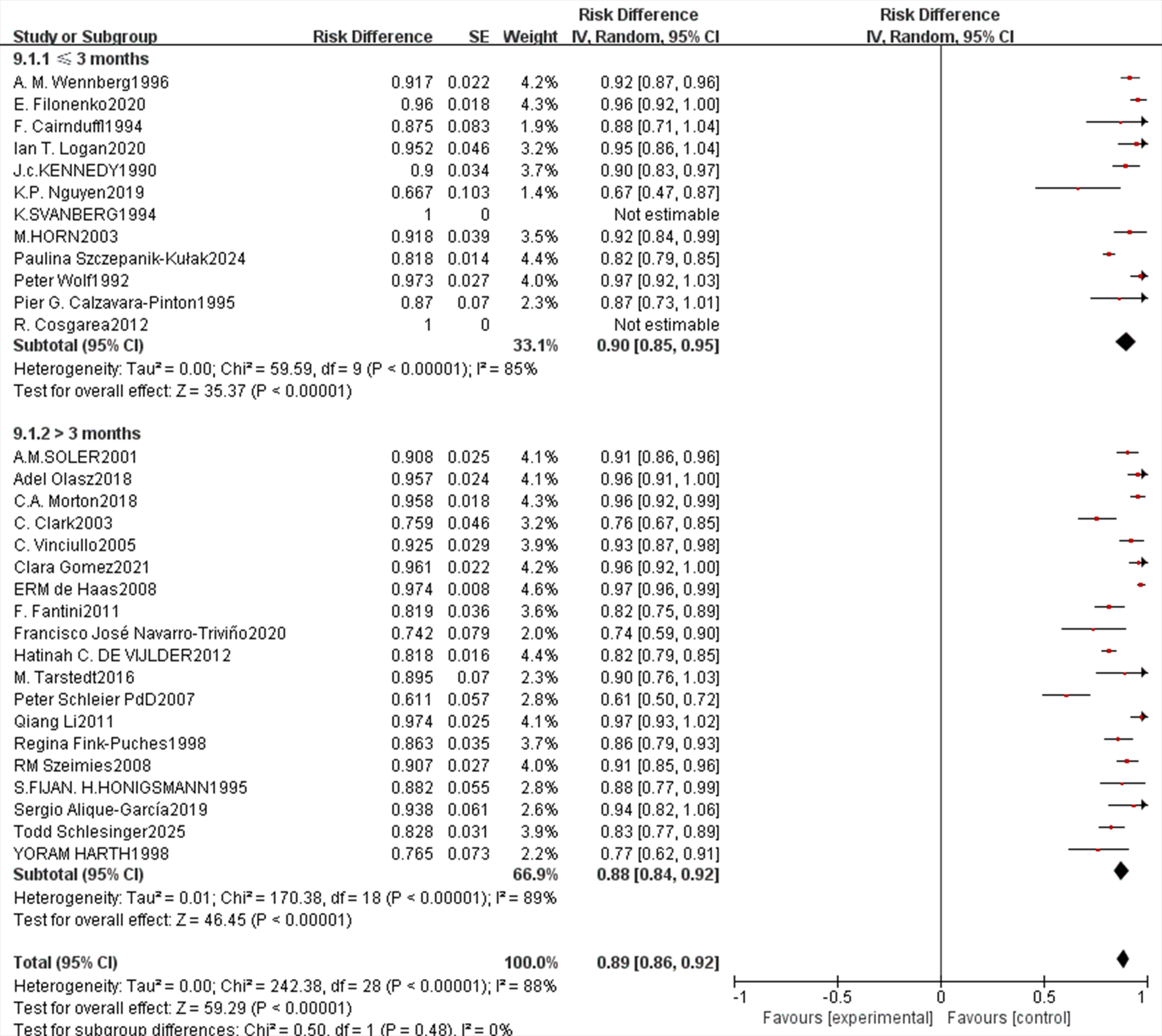


Supplementary Picture 15 Forest plot of CR rate for superficial basal cell carcinoma stratified by Follow-up period.


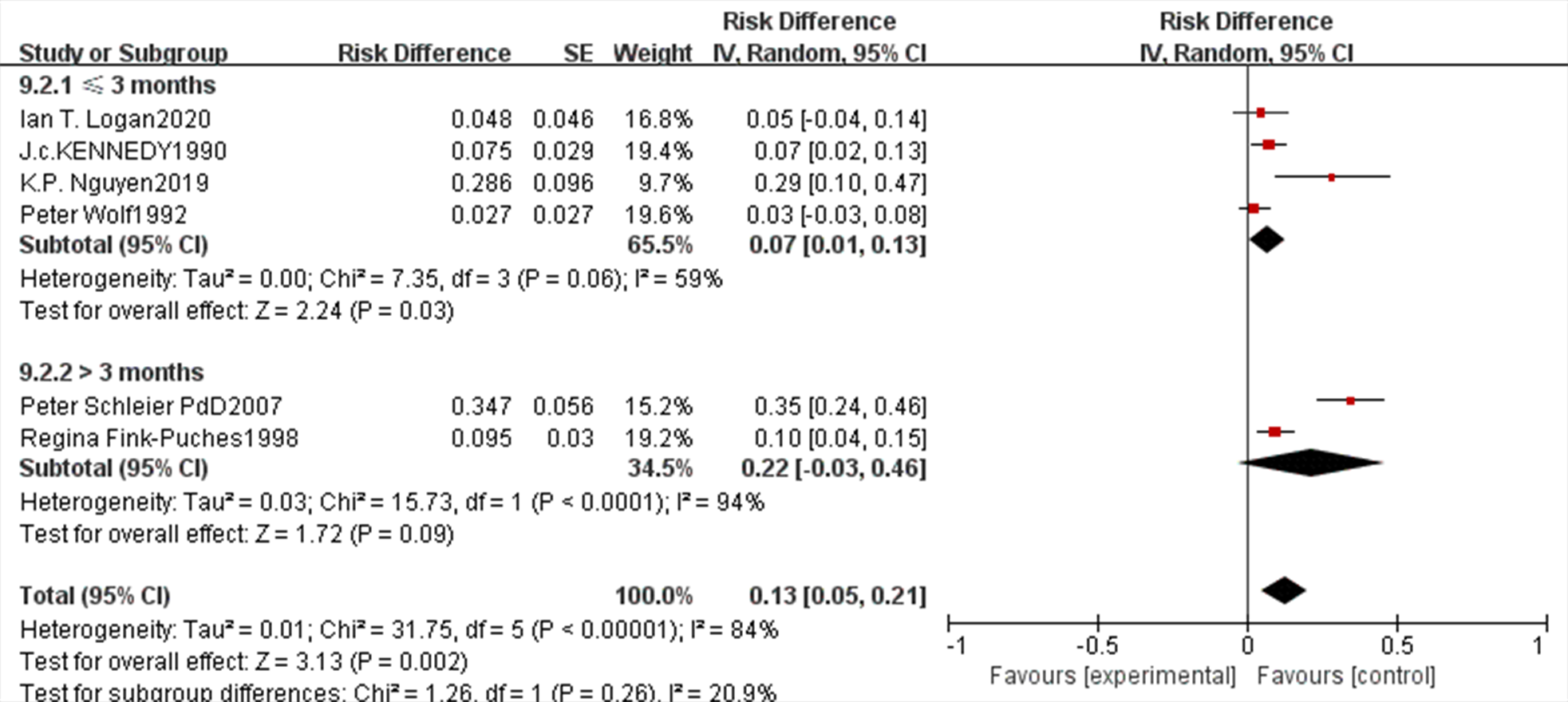


Supplementary Picture 16 Forest plot of PR rate for superficial basal cell carcinoma stratified by Follow-up period.


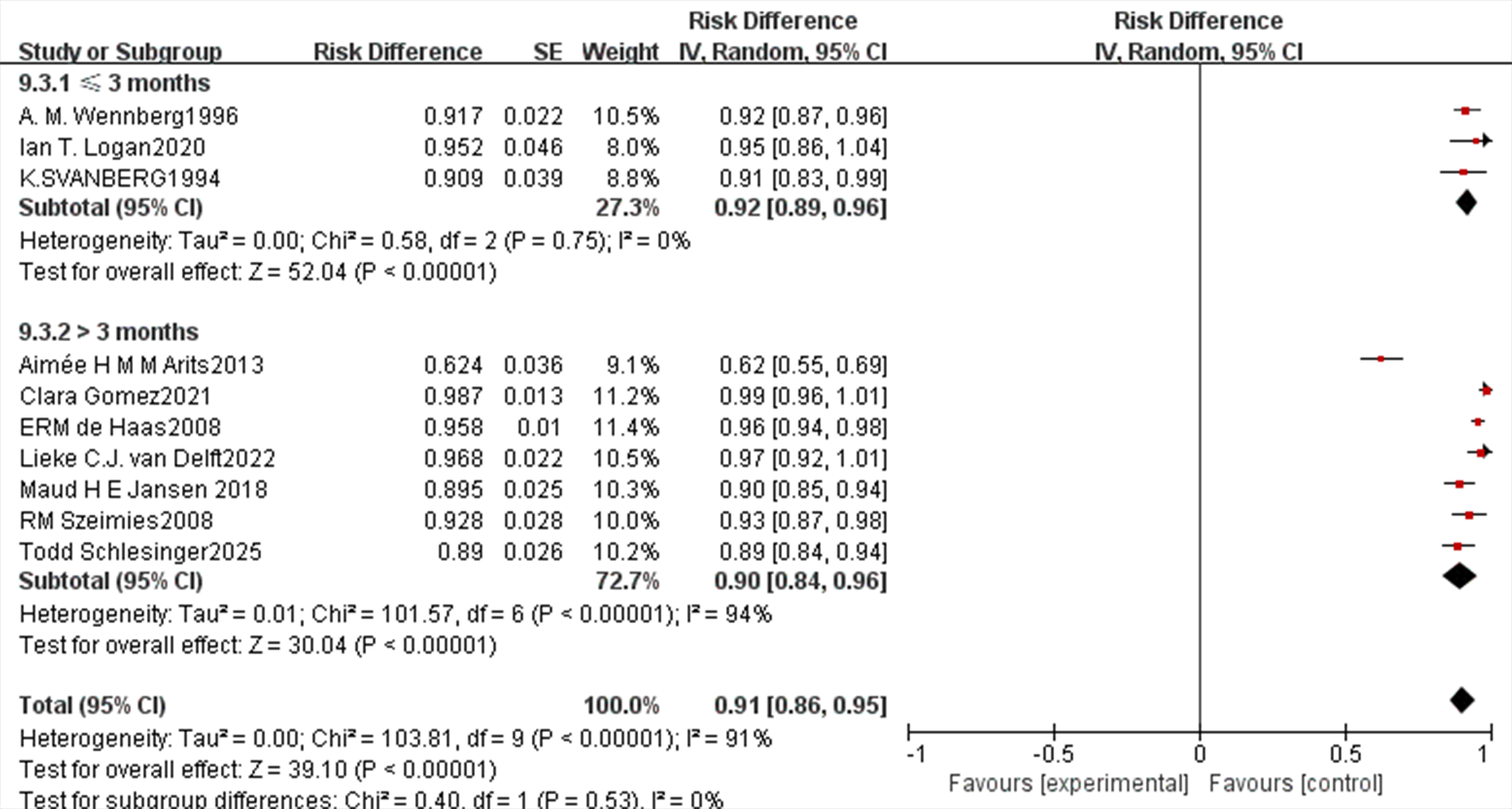


Supplementary Picture 17 Forest plot of Beauty effect rate for superficial basal cell carcinoma stratified by Follow-up period.


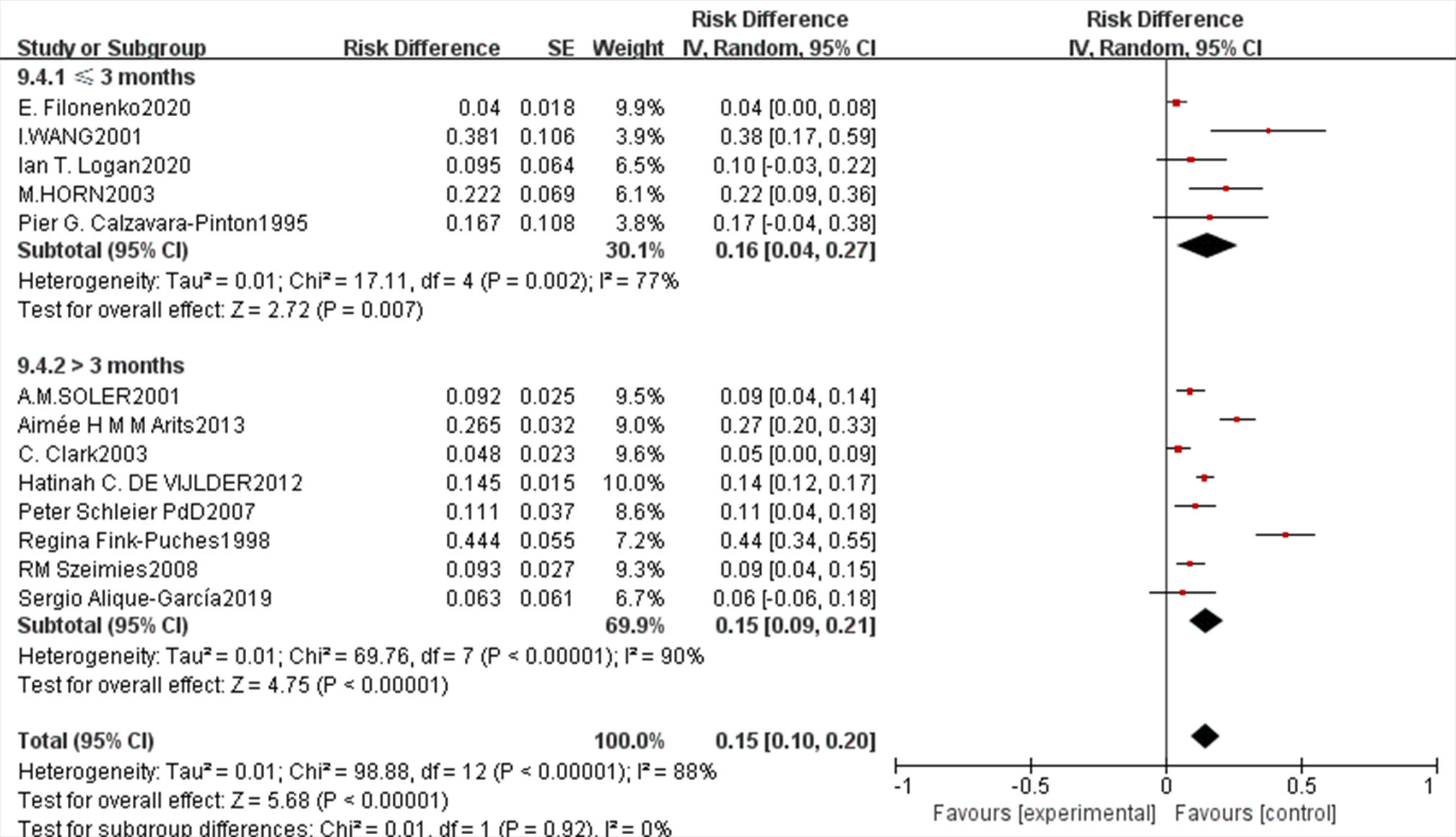


Supplementary Picture 18 Forest plot of Recurrent probability for superficial basal cell carcinoma stratified by Follow-up period.


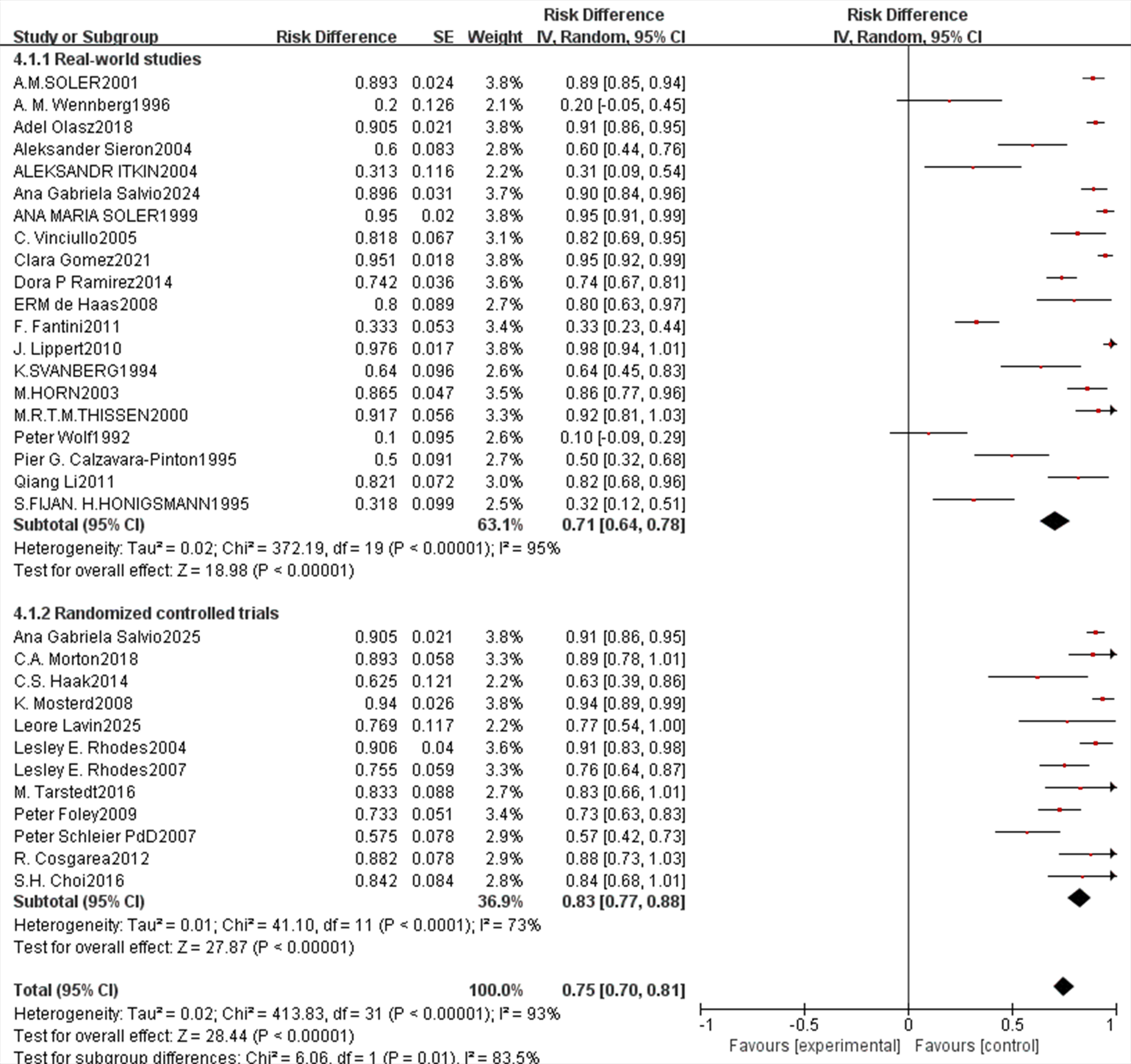


Supplementary Picture 19 Forest plot of CR rate for nodular basal cell carcinoma stratified by study design.


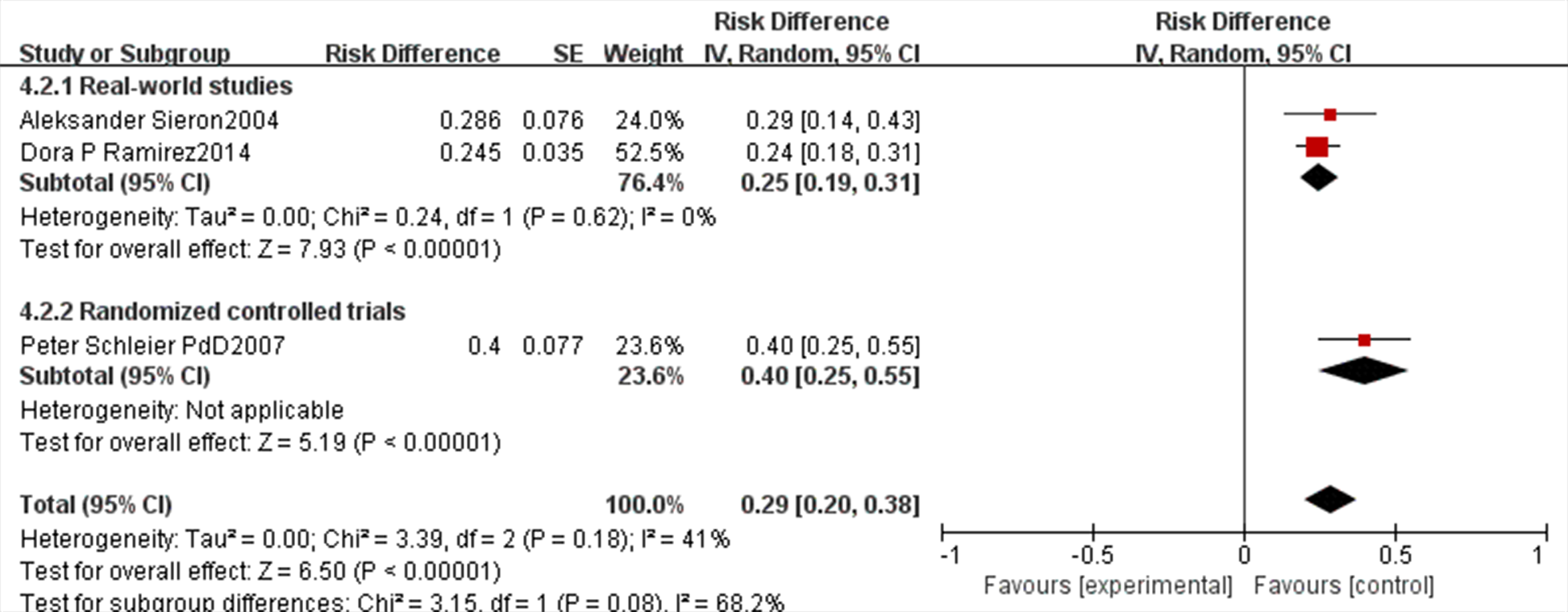


Supplementary Picture 20 Forest plot of PR rate for nodular basal cell carcinoma stratified by study design.


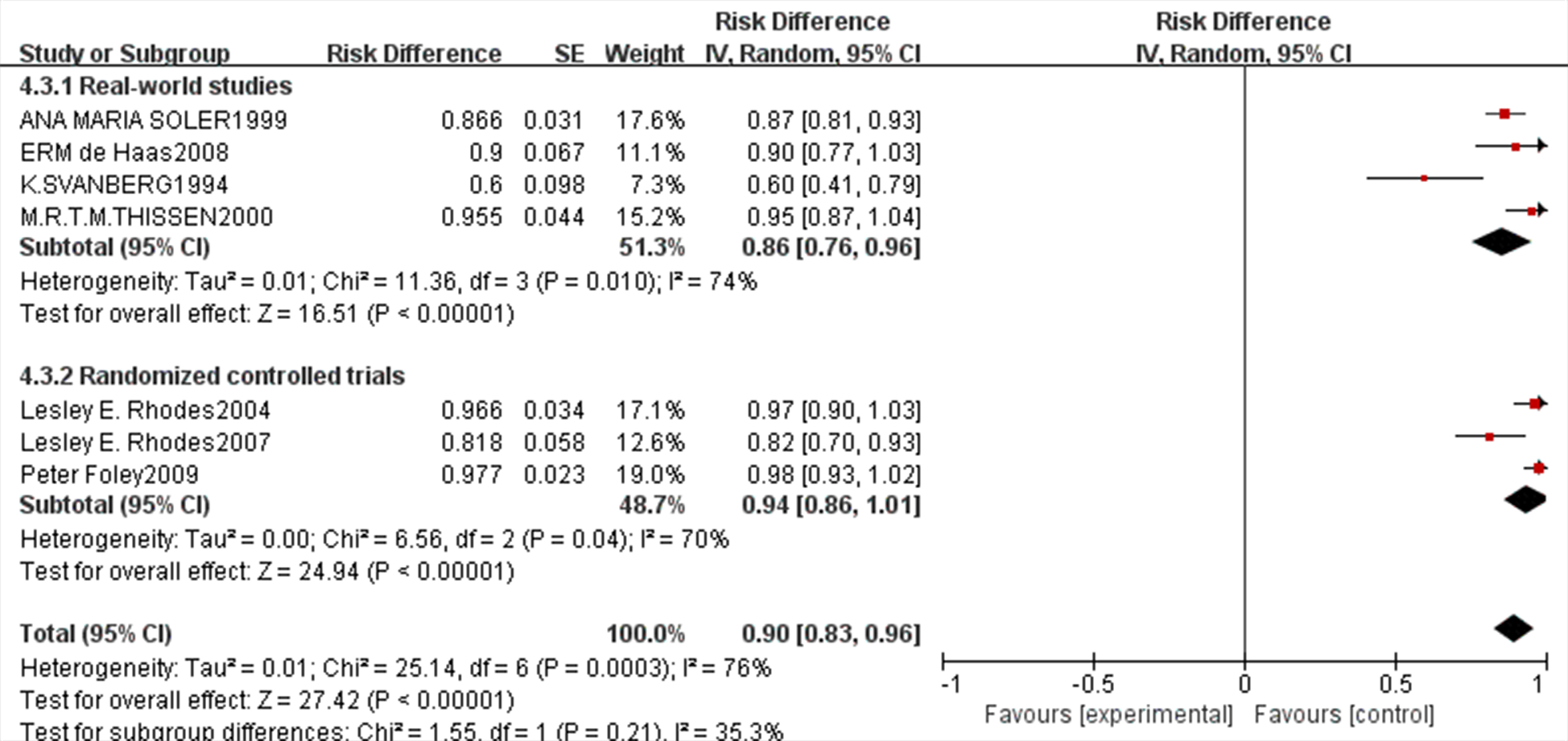


Supplementary Picture 21 Forest plot of Beauty effect rate for nodular basal cell carcinoma stratified by study design.


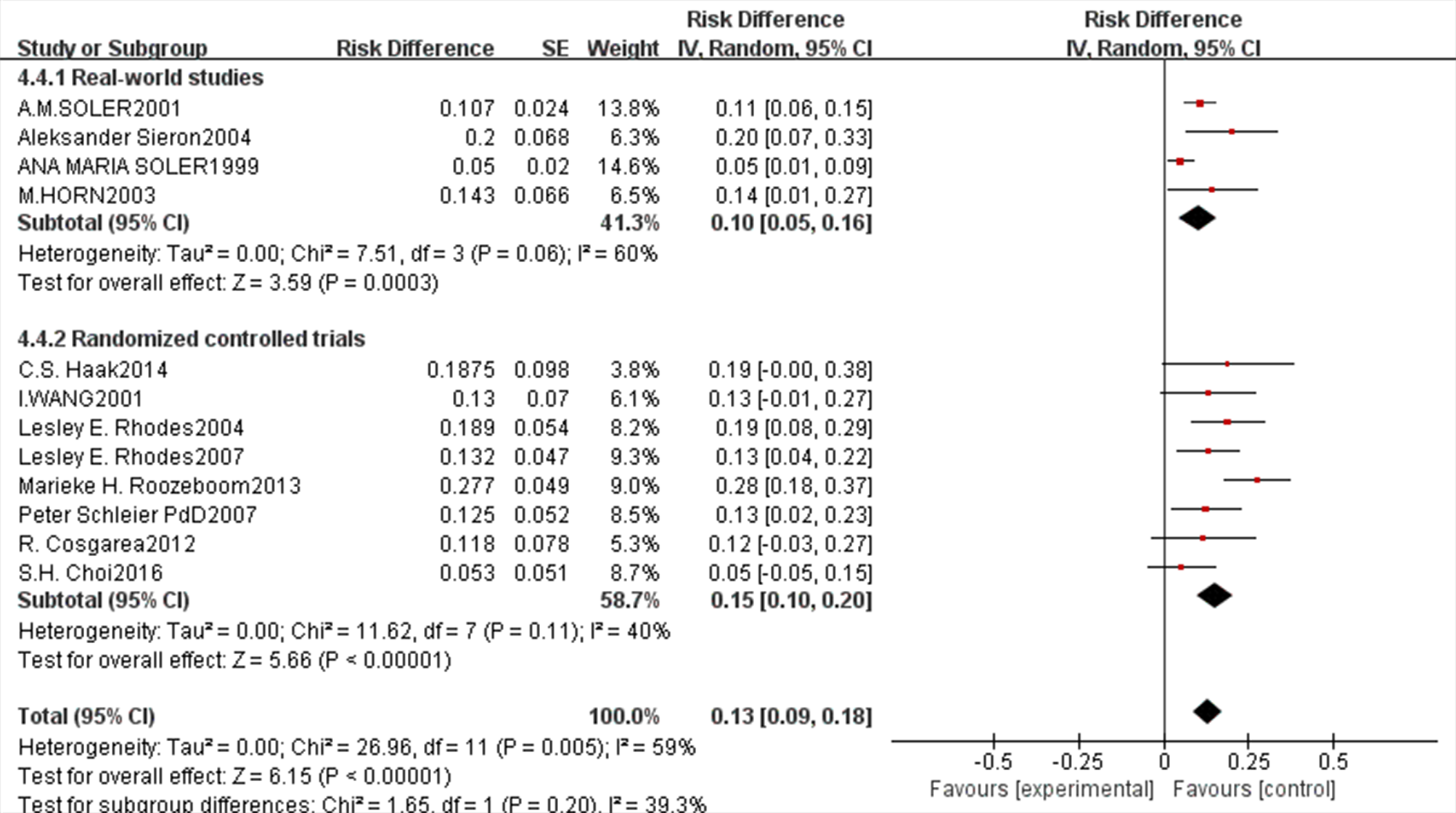


Supplementary Picture 22 Forest plot of Recurrent probability for nodular basal cell carcinoma stratified by study design.


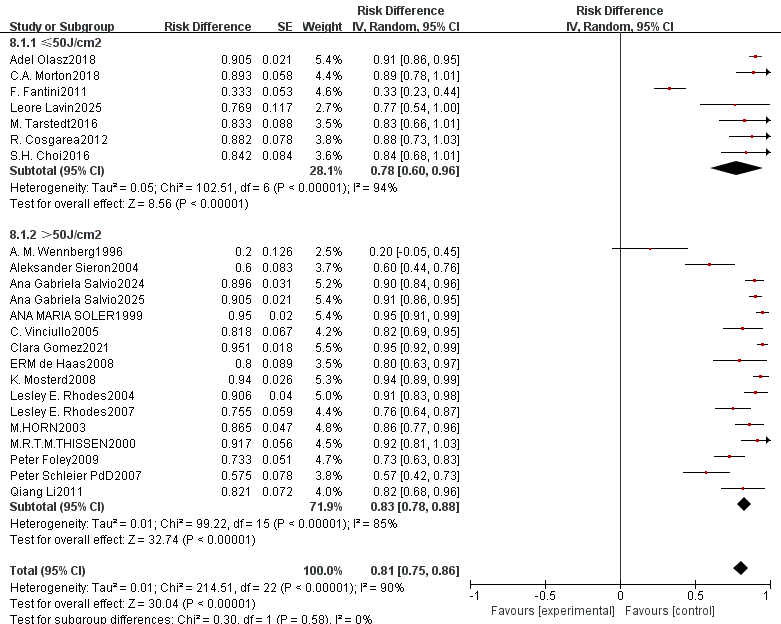


Supplementary Picture 23 Forest plot of CR rate for nodular basal cell carcinoma stratified by Light dose.


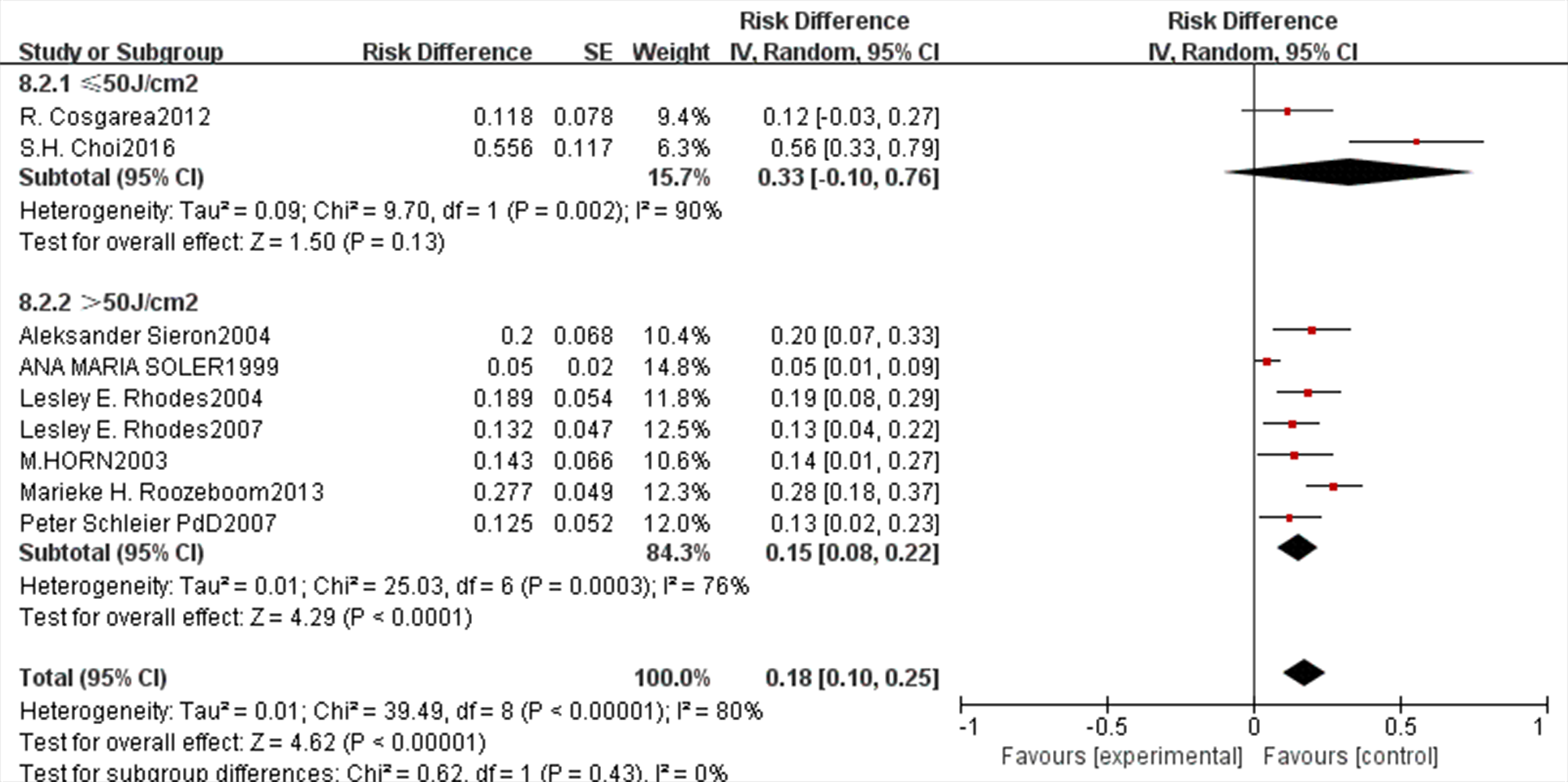


Supplementary Picture 24 Forest plot of Recurrent probability for nodular basal cell carcinoma stratified by Light dose.


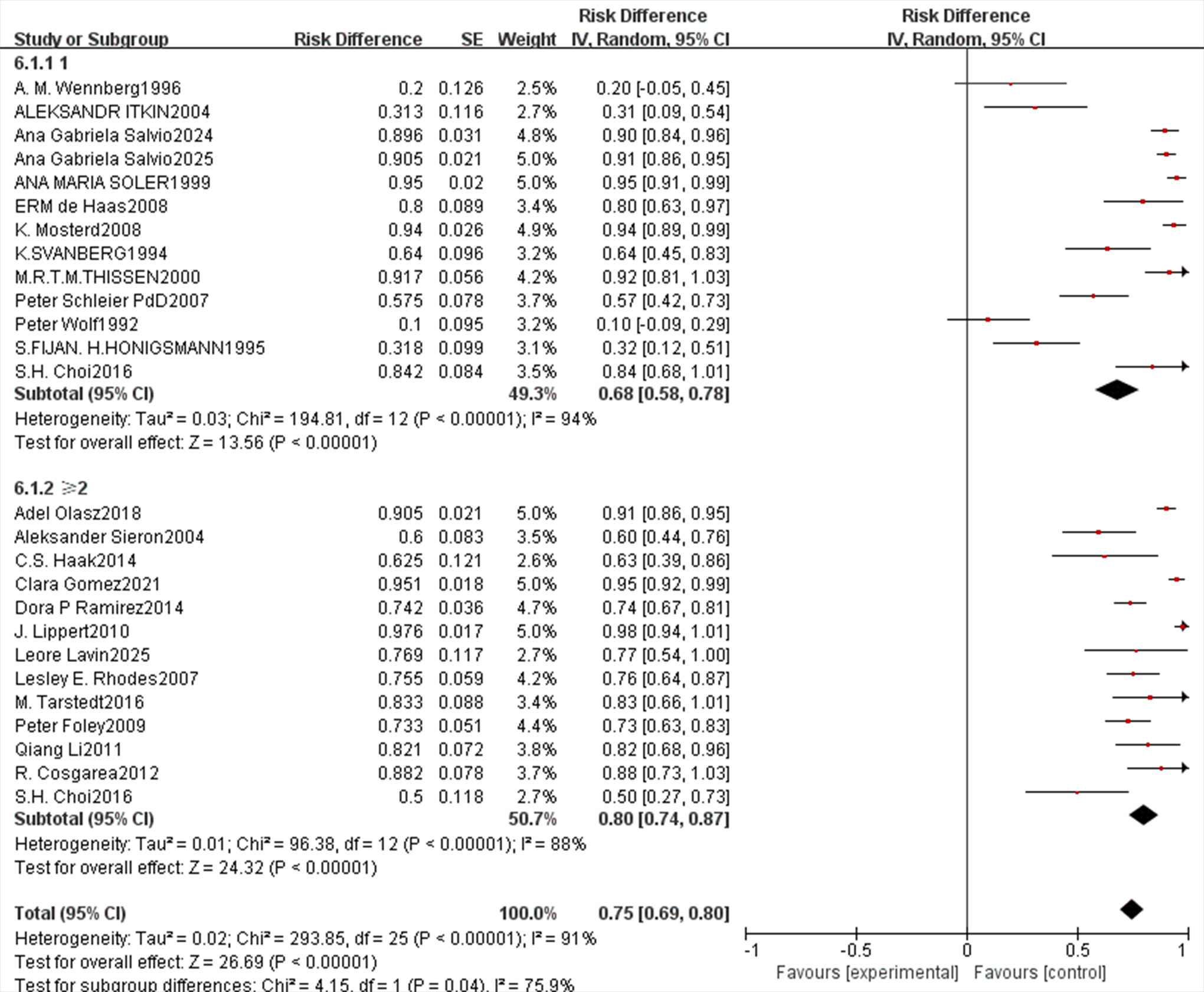


Supplementary Picture 25 Forest plot of CR rate for nodular basal cell carcinoma stratified by Number of treatments.


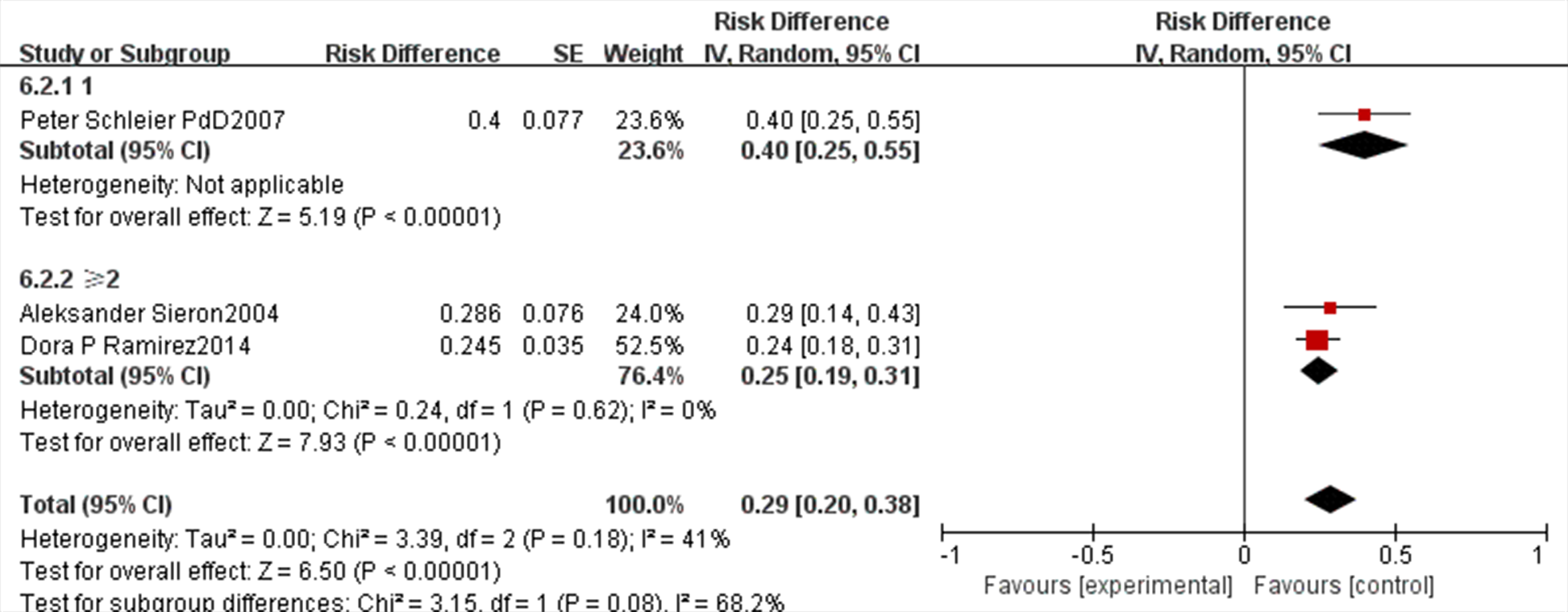


Supplementary Picture 26 Forest plot of PR rate for nodular basal cell carcinoma stratified by Number of treatments.


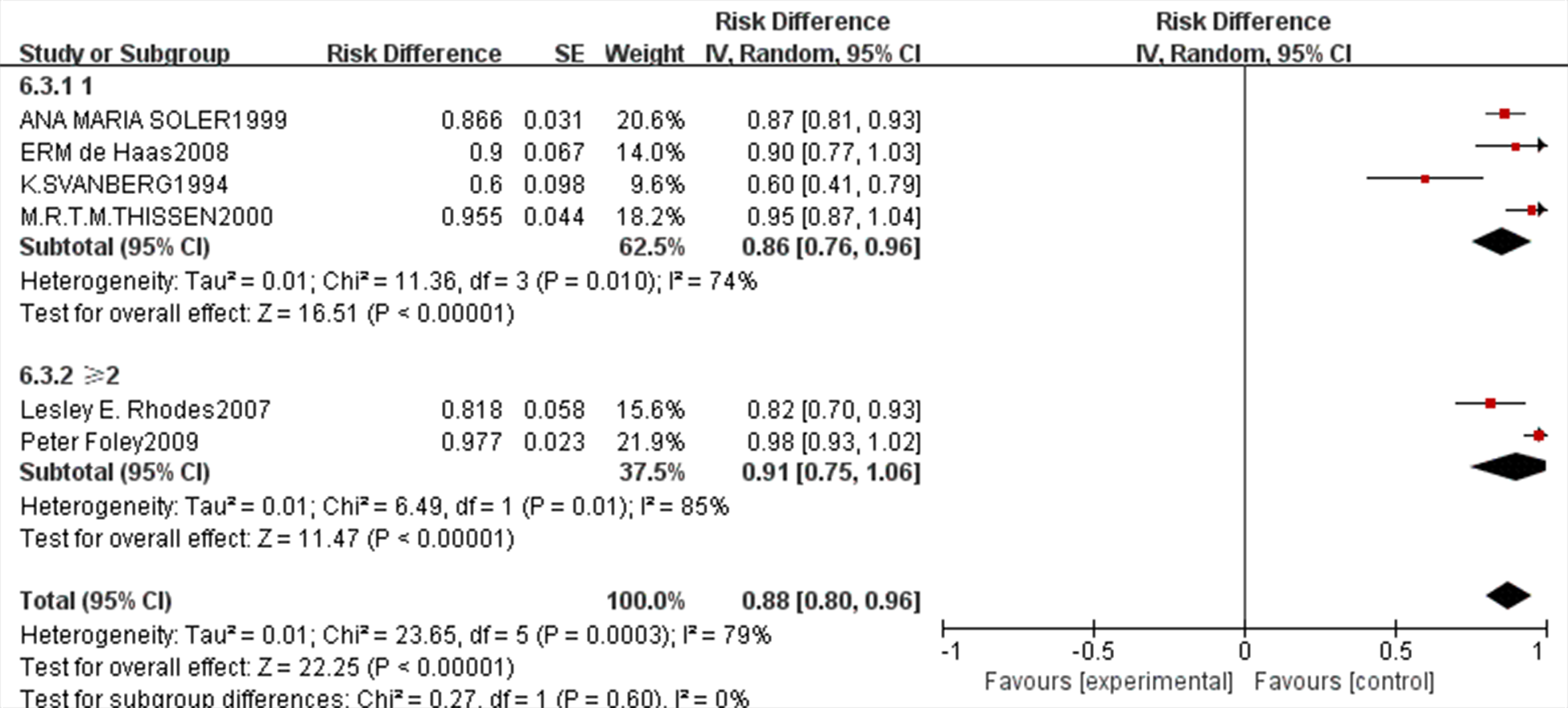


Supplementary Picture 27 Forest plot of Beauty effect rate for nodular basal cell carcinoma stratified by Number of treatments.


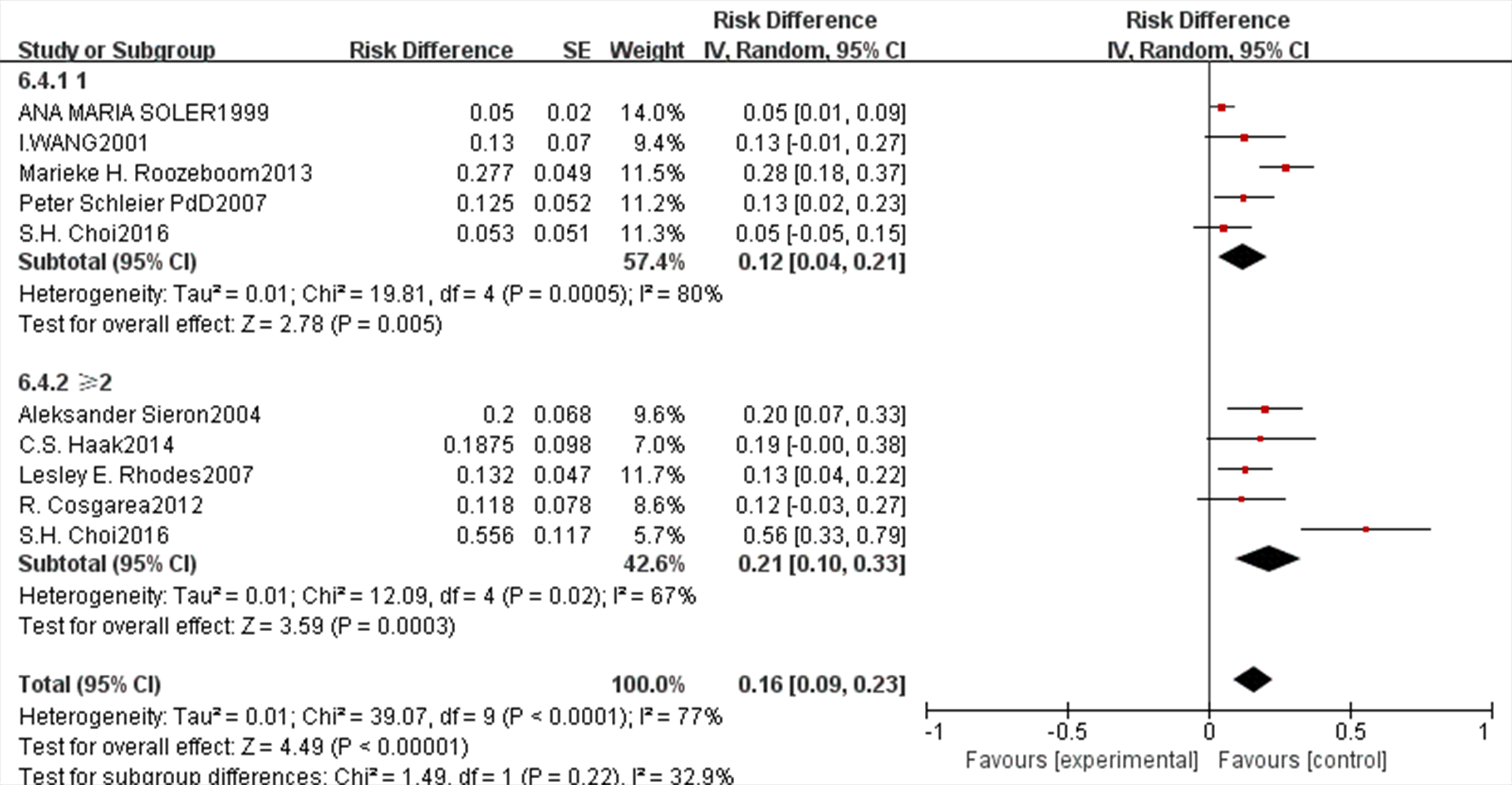


Supplementary Picture 28 Forest plot of Recurrent probability for nodular basal cell carcinoma stratified by Number of treatments.


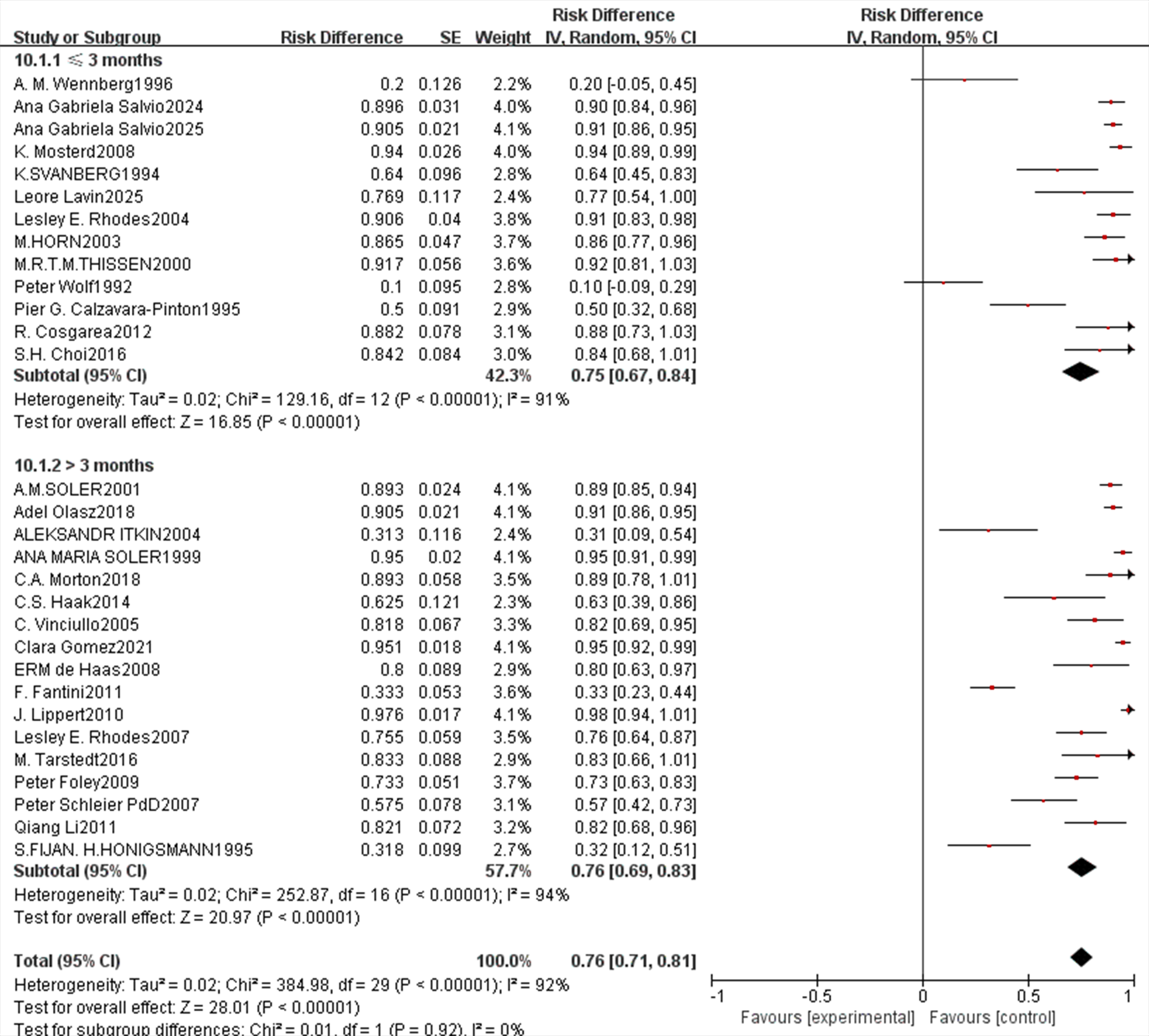


Supplementary Picture 29 Forest plot of CR rate for nodular basal cell carcinoma stratified by Follow-up period.


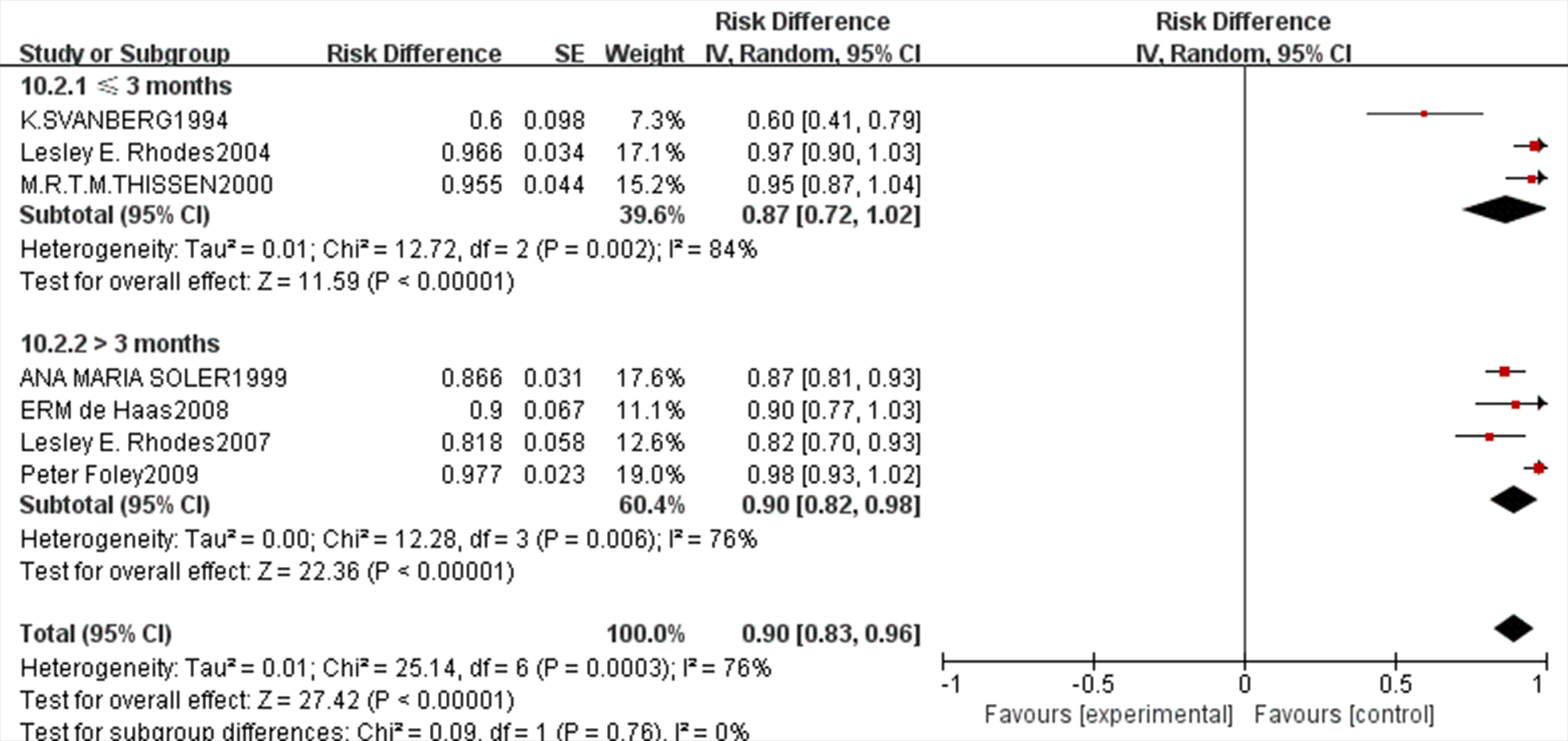


Supplementary Picture 30 Forest plot of Beauty effect rate for nodular basal cell carcinoma stratified by Follow-up period.


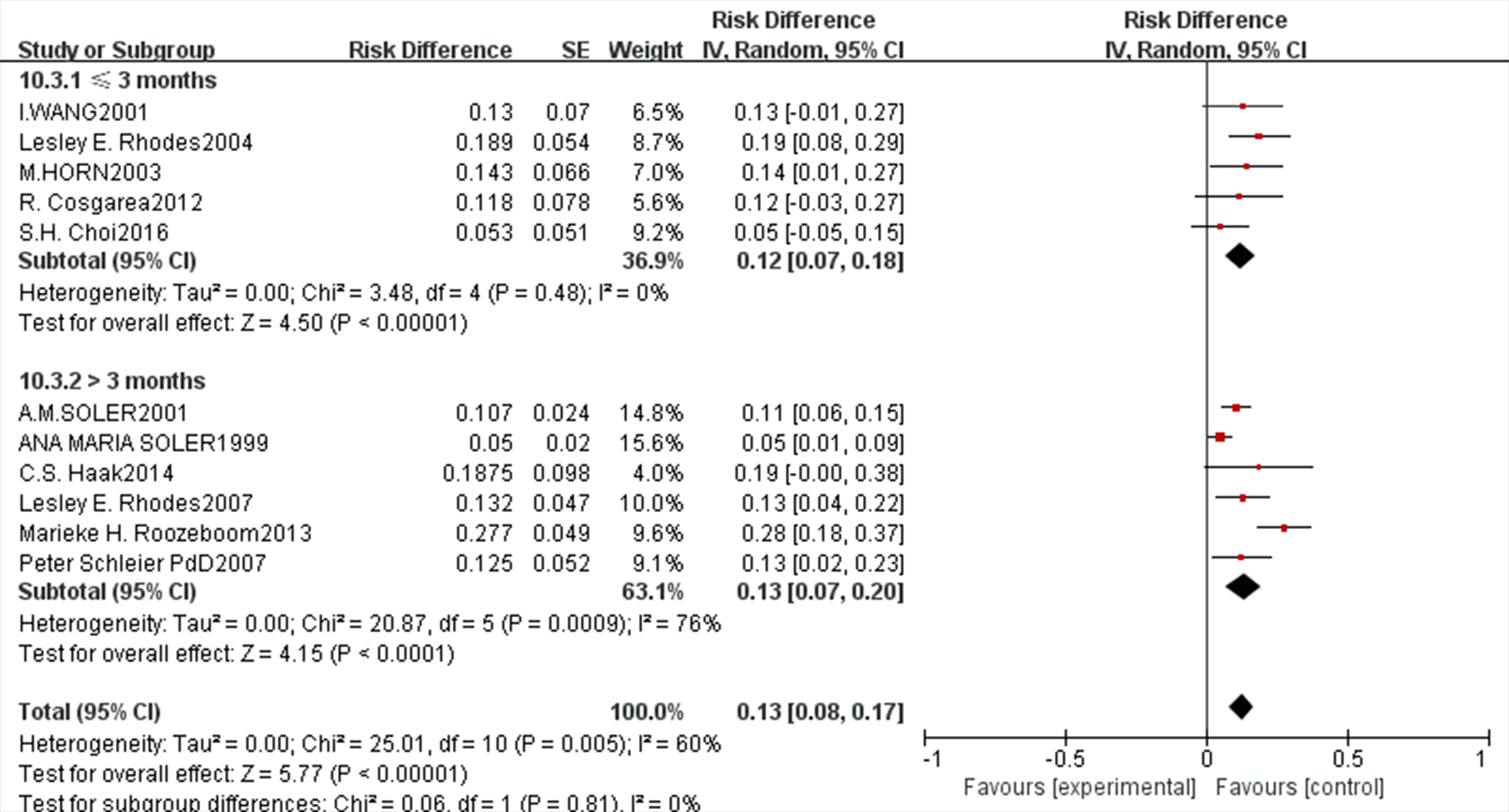


Supplementary Picture 31 Forest plot of Recurrent probability for nodular basal cell carcinoma stratified by Follow-up period.
