# Supplementary material for: Efficacy and safety of ALA/MLA photodynamic therapy for superficial and nodular basal cell carcinoma: a systematic review and meta-analysis
Source: Front Oncol. 2026 Apr 14;16:1802984. doi: 10.3389/fonc.2026.1802984 (PMC13121321; doi:10.3389/fonc.2026.1802984)
Supplement: Supplementary file 2 [file Table1.docx]

**Supplementary Table 1** Assessment of non-randomized controlled trials in the version of MINORS.

| Study | A clearly  stated  aim | Inclusion of  consecutive  patients | Prospective  collection of  data | Endpoint  appropriate  to the study  aim | Unbiased  assessment  of endpoints | Follow-up  period  appropriate  to the major  endpoint | Loss to  follow  up not  exceeding  5% | Prospective  calculation  of the study  size | Total score |
| --- | --- | --- | --- | --- | --- | --- | --- | --- | --- |
| J.c.KENNEDY1990 | 2 | 2 | 2 | 2 | 1 | 2 | 2 | 1 | 14 |
| Peter Wolf1992 | 2 | 2 | 2 | 2 | 2 | 2 | 2 | 2 | 16 |
| F. Cairnduffl1994 | 2 | 2 | 2 | 1 | 2 | 2 | 2 | 1 | 14 |
| K.SVANBERG1994 | 2 | 2 | 2 | 2 | 1 | 2 | 2 | 1 | 14 |
| Pier G. Calzavara-Pinton1995 | 2 | 2 | 2 | 2 | 2 | 2 | 1 | 2 | 15 |
| S.FIJAN. H.HONIGSMANN1995 | 2 | 2 | 2 | 1 | 1 | 2 | 2 | 1 | 13 |
| P.J.N. MEIJNDERS1996 | 2 | 2 | 2 | 2 | 2 | 2 | 2 | 2 | 16 |
| A. M. Wennberg1996 | 2 | 2 | 2 | 1 | 2 | 2 | 0 | 2 | 13 |
| Regina Fink-Puches1998 | 2 | 2 | 2 | 2 | 2 | 2 | 2 | 2 | 16 |
| YORAM HARTH1998 | 2 | 2 | 2 | 2 | 2 | 2 | 2 | 1 | 15 |
| A.F. Hürlimann1998 | 2 | 2 | 2 | 1 | 2 | 2 | 2 | 2 | 15 |
| A.M.SOLER2001 | 2 | 2 | 2 | 2 | 1 | 2 | 2 | 1 | 14 |
| I.WANG2001 | 2 | 2 | 2 | 2 | 2 | 2 | 2 | 2 | 16 |
| C. Clark2003 | 2 | 2 | 2 | 2 | 2 | 2 | 1 | 2 | 15 |
| M.HORN2003 | 2 | 2 | 2 | 2 | 2 | 1 | 2 | 1 | 14 |
| Aleksander Sieron2004 | 2 | 2 | 2 | 2 | 2 | 2 | 2 | 2 | 16 |
| C. Vinciullo2005 | 2 | 2 | 2 | 2 | 2 | 2 | 2 | 2 | 16 |
| Willem M. STAR2006 | 2 | 2 | 2 | 2 | 2 | 2 | 0 | 2 | 14 |
| Peter Schleier PdD2007 | 2 | 2 | 2 | 2 | 2 | 2 | 2 | 2 | 16 |
| ERM de Haas2008 | 2 | 2 | 2 | 2 | 2 | 2 | 2 | 2 | 16 |
| RM Szeimies2008 | 2 | 2 | 2 | 2 | 2 | 2 | 1 | 1 | 14 |
| F. Fantini2011 | 2 | 2 | 2 | 2 | 1 | 2 | 2 | 2 | 15 |
| Qiang Li2011 | 2 | 2 | 2 | 2 | 2 | 2 | 2 | 2 | 16 |
| Hatinah C. DE VIJLDER2012 | 2 | 2 | 2 | 1 | 2 | 2 | 2 | 2 | 15 |
| R. Cosgarea2012 | 2 | 2 | 2 | 2 | 2 | 2 | 2 | 1 | 15 |
| Aimée H M M Arits2013 | 2 | 2 | 2 | 2 | 2 | 2 | 2 | 2 | 16 |
| Dora P Ramirez2014 | 2 | 2 | 2 | 1 | 2 | 2 | 1 | 1 | 13 |
| Marieke H. Roozeboom2016 | 2 | 2 | 2 | 2 | 2 | 2 | 1 | 2 | 15 |
| M. Tarstedt 2016 | 2 | 2 | 2 | 2 | 2 | 2 | 1 | 1 | 14 |
| Maud H E Jansen 2018 | 2 | 2 | 2 | 1 | 2 | 2 | 2 | 2 | 15 |
| C.A. Morton2018 | 2 | 2 | 2 | 1 | 2 | 2 | 2 | 2 | 15 |
| Adel Olasz 2018 | 2 | 2 | 2 | 2 | 2 | 2 | 2 | 2 | 16 |
| Sergio Alique-García2019 | 2 | 2 | 2 | 2 | 2 | 2 | 2 | 1 | 15 |
| K.P. Nguyen2019 | 2 | 2 | 2 | 2 | 2 | 2 | 2 | 2 | 16 |
| E. Filonenko2020 | 2 | 2 | 2 | 2 | 1 | 1 | 2 | 1 | 13 |
| Ian T. Logan2020 | 2 | 2 | 2 | 2 | 2 | 2 | 2 | 2 | 16 |
| Francisco José Navarro-Triviño2020 | 2 | 2 | 2 | 2 | 1 | 2 | 2 | 2 | 15 |
| Clara Gomez2021 | 2 | 2 | 2 | 1 | 2 | 2 | 2 | 2 | 15 |
| Lieke C.J. van Delft2022 | 2 | 2 | 2 | 2 | 2 | 1 | 1 | 2 | 14 |
| Paulina Szczepanik-Kułak2024 | 2 | 2 | 2 | 2 | 2 | 2 | 2 | 2 | 16 |
| Todd Schlesinger 2025 | 2 | 2 | 2 | 2 | 1 | 2 | 2 | 1 | 14 |
| ANA MARIA SOLER1999 | 2 | 2 | 2 | 2 | 2 | 2 | 2 | 1 | 15 |
| M.R.T.M.THISSEN2000 | 2 | 2 | 2 | 2 | 1 | 2 | 2 | 2 | 15 |
| ALEKSANDR ITKIN2004 | 2 | 2 | 2 | 2 | 2 | 2 | 2 | 1 | 15 |
| Lesley E. Rhodes2004 | 2 | 2 | 2 | 2 | 1 | 2 | 2 | 1 | 14 |
| Lesley E. Rhodes2007 | 2 | 2 | 2 | 2 | 1 | 2 | 2 | 1 | 14 |
| K. Mosterd 2008 | 2 | 2 | 2 | 2 | 1 | 2 | 2 | 2 | 15 |
| Peter Foley 2009 | 2 | 2 | 2 | 2 | 2 | 2 | 2 | 1 | 15 |
| J. Lippert2010 | 2 | 2 | 2 | 2 | 1 | 2 | 2 | 2 | 15 |
| Marieke H. Roozeboom2013 | 2 | 2 | 2 | 2 | 1 | 2 | 2 | 1 | 14 |
| C.S. Haak2014 | 2 | 2 | 2 | 2 | 1 | 2 | 2 | 2 | 15 |
| S.H. Choi2016 | 2 | 2 | 2 | 2 | 2 | 2 | 2 | 1 | 15 |
| Ana Gabriela Salvio2024 | 2 | 2 | 2 | 2 | 2 | 2 | 2 | 1 | 15 |
| Ana Gabriela Salvio2025 | 2 | 2 | 2 | 2 | 2 | 2 | 2 | 2 | 16 |
| Leore Lavin2025 | 2 | 2 | 2 | 2 | 2 | 2 | 2 | 2 | 16 |
